# Paeoniae Radix Rubra can enhance fatty acid β-oxidation and alleviate gut microbiota disorder in α-naphthyl isothiocyanate induced cholestatic model rats

**DOI:** 10.3389/fphar.2022.1002922

**Published:** 2022-10-21

**Authors:** Jing-Jing Xu, Feng Xu, Wei Wang, Peng-Pu Wang, Jing Xian, Xing Han, Ming-Ying Shang, Guang-Xue Liu, Xuan Wang, Shao-Qing Cai

**Affiliations:** State Key Laboratory of Natural and Biomimetic Drugs, School of Pharmaceutical Sciences, Peking University, Beijing, China

**Keywords:** cholestasis, fatty acid β-oxidation, gut microbiota, Paeoniae Radix Rubra, pharmacodynamic mechanisms

## Abstract

Cholestasis is the most destructive pathological manifestation of liver disease and available treatments are very limited. Paeoniae Radix Rubra (PRR) is an important traditional Chinese drug used to treat cholestasis. This study combined targeted metabonomics, PCR array analysis, and 16S rRNA sequencing analysis to further clarify the mechanisms of PRR in the treatment of cholestasis. PRR conspicuously reversed the elevation of fatty acids (FFA 14:0 and other 14 fatty acids) and the decrease of organic acids (pyruvic acid and citric acid) in a cholestatic model induced by α-naphthyl isothiocyanate (ANIT). Eight elevated amino acids (L-proline, etc.) and five elevated secondary bile acids (taurohyodeoxycholic acid, etc.) in model rats were also reduced by PRR. Pathway analysis revealed that PRR significantly alleviated eight pathways (β-alanine metabolism). Furthermore, we found that PRR significantly reversed the decrease of Cpt1a, Hadha, Ppara, and Slc25a20 (four genes relevant to fatty acid β-oxidation) mRNAs caused by ANIT, and PRR conspicuously decreased nine acylcarnitines (the forms of fatty acids into mitochondria for β-oxidation) that increased in model rats. These results indicate that PRR could enhance fatty acid β-oxidation, which may be the way for PRR to reduce the levels of 15 fatty acids in the serum of model rats. 16S rRNA sequencing analysis revealed that PRR alleviated gut microbiota disorders in model rats, including upregulating four genera (*Coprococcus*, *Lactobacillus*, etc.) and downregulating four genera (*Bacteroides*, *Escherichia*, etc.). As the relative abundance of these eight genera was significantly correlated with the levels of the five secondary bile acids (deoxycholic acid, taurolithocholic acid, etc.) reduced by PRR, and *Bacteroides* and *Escherichia* were reported to promote the production of secondary bile acid, we inferred that the downregulation of PRR on five secondary bile acids in model rats was inseparable from gut microbiota. Thus, the gut microbiota also might be a potential pharmacological target for the anticholestatic activity of PRR. In conclusion, we consider that the mechanisms of PRR in treating cholestasis include enhancing fatty acid β-oxidation and alleviating gut microbiota disorders.

## 1 Introduction

Cholestasis is a clinical syndrome caused by abnormal bile synthesis and secretion, as well as mechanical or functional obstacles to bile flow in the intrahepatic and extrahepatic bile ducts, which impede the normal flow of bile components into the duodenum and back into the blood circulation. Cholestasis is the most destructive pathological manifestation of a variety of infectious (hepatitis A, hepatitis B, etc.) and non-infectious (alcoholic liver disease, primary biliary cholangitis, and primary sclerosing cholangitis, etc.) liver diseases (Jiang et al., 2021). The accumulated bile will damage the liver tissue and ultimately lead to the development of liver failure and biliary cirrhosis; the appearance of cholestasis often indicates a poor prognosis (Chascsa et al., 2017). Currently, the drugs used to treat cholestasis are very limited. Ursodeoxycholic acid (UDCA) is a first-line drug approved by the United States Food and Drug Administration for the treatment of cholestasis. However, approximately 20%–40% of patients do not respond or respond poorly to treatment. Furthermore, high-dose UDCA treatment will increase the risk of liver cirrhosis, esophageal varices, and cholangiocarcinoma (Carbone et al., 2013). Thus, the development of novel therapeutic reagents for cholestasis is of utmost importance.

Paeoniae Radix Rubra (PRR), derived from the dried roots of *Paeonia lactiflora* Pall. or *Paeonia veitchii* Lynch., is an important and common traditional Chinese drug (TCD) used to treat liver diseases (State Pharmacopoeia Committee of the People’s Republic of China, 2020). Clinical and experimental studies have demonstrated that PRR can effectively treat cholestasis. For example, Cheng-bai Wang, a prominent integrative medicine hepatologist, considered “cooling blood and activating blood circulation” as the main therapeutic principle and used high-dose PRR (300 g/d) to treat cholestasis, achieving remarkable results (Wang, 2011). Chidan Tuihuang Granule with PRR as the main drug in Phase III clinical trial for the treatment of cholestasis had an efficacy of 88.66% (He, 2003). Wei et al. (2011) investigated the pharmacodynamic effects of PRR at different doses (1, 9, 18, 36, 54, 72, and 81 g/(kg·d) PRR extract) in ANIT induced cholestatic rats and found that PRR could significantly alleviate cholestasis in a dose-dependent manner.

Studies on the pharmacodynamic mechanisms of PRR in the treatment of cholestasis have been conducted from the perspective of anti-oxidation and anti-inflammation and revealed that PRR may alleviate cholestasis by activating Nrf2 *via* the PI3K/Akt signaling pathway and by regulating the NF-κB-NLRP3 inflammasome pathway (Ma et al., 2015; Ma et al., 2018). However, these studies are not sufficient to elucidate the multi-target and multi-path action mechanisms of PRR.

The systemic features of metabonomics are consistent with the holistic characteristic of TCDs, and has become a powerful technology to explore the pharmacodynamic mechanisms of TCDs. Ma et al. (2016) conducted an untargeted metabonomics study and discovered that PRR could significantly reverse the abnormal changes of five amino acids (L-palmitoylcarnitine, 4-guanidinobutanoate, pantothenate, D-arginine, and 2-phenylacetamide) and three bile acids (glycocholic acid, glycochendeoxycholic acid, and taurocholic acid) in the ANIT-induced cholestatic rat model. Untargeted metabonomics is unable to accurately define the structures of metabolites. Thus, it is necessary to apply a targeted metabonomics analysis, which is an accurate qualitative analysis for metabolites and with better reproducibility and higher sensitivity than untargeted metabonomics, to find more differential metabolites with definite structures.

Recently, the ANIT-induced cholestatic rat model, which is pathologically similar to human intrahepatic cholestasis, has been widely used in related researches (Waters et al., 2001). Several reports have revealed that the levels of fatty acids are significantly elevated and the levels of organic acids are significantly reduced in the serum of the ANIT-induced cholestatic rat model by untargeted and targeted metabonomics analyses (Waters et al., 2001; Liu et al., 2021). However, there have been no reports about investigating whether PRR can alleviate the disorders of fatty acids and organic acids induced by cholestasis.

Therefore, in this study, we first combined targeted metabonomics with multivariate statistical analysis to explore changes in the levels of serum fatty acids, bile acids, amino acids, and organic acids in cholestatic model rats induced by ANIT. Next, the main regulatory effects of PRR treatment on these metabolic disorders were explored. Based on the results obtained by metabonomics, PCR array and 16S rRNA sequencing analyses were performed to explore how PRR regulated key metabolites to further elucidate the pharmacodynamic mechanisms of PRR in treating cholestasis.

## 2 Methods

### 2.1 Materials

ANIT (Lot no. STBH7289) and UDCA (Lot no. STBH3896) were purchased from Sigma-Aldrich (St. Louis, MO, United States). High-performance liquid chromatography (HPLC) grade methanol and acetonitrile were purchased from Merck Group (Darmstadt, Germany) and HPLC grade formic acid was obtained from Fisher Scientific Corporation (Loughborough, United Kingdom). Ultra-high purity water (18.2 MΩ, total organic carbon <5 ppb) was prepared using a Millipore Milli-Q Integral 3 Ultrapure Water System (Billerica, MA, United States). TRIzol (Lot no. RE30213601) was purchased from Coolaber Science and Technology Corporation (Beijing, China). PRR was obtained from the Beijing Tianheng Pharmacy (Beijing, China; Lot no. 140701) on 21 May 2019, and its sample was authenticated as dried roots of *Paeonia lactiflora* Pall. (Ranunculaceae) by Dr. Feng Xu (School of Pharmaceutical Sciences, Peking University). A voucher sample (No. 7838) was deposited at the Herbarium of Pharmacognosy, School of Pharmaceutical Sciences, Peking University.

### 2.2 Preparation of PRR extract

Freeze-dried powder of PRR extract (360.58 g) was prepared from 1 kg PRR (the dried roots of *Paeonia lactiflora* Pall.), using a previously described method (Liang et al., 2013). The extraction ratio is 36.06%.

The UPLC fingerprint of the PRR extract is presented in Supplementary Information S1. The contents of five constituents (paeoniflorin, catechin, oxypaeoniflorin, benzoylpaeoniflorin, and gallic acid) in the PRR extract were also determined and listed in Supplementary Information S2.

### 2.3 Animals and experimental design

Fifty-two male Sprague-Dawley rats (180–200 g) were purchased from the Experimental Animal Center of Peking University Health Science Center (Beijing, China). First, forty rats were randomly divided into four groups (control, model, PRR, and UDCA groups) and used to conduct the pharmacodynamic experiment; then, twenty-four rats of them (eight rats were randomly selected from control group, model group, and PRR group, respectively) were used for the targeted metabonomics study and gut microbiota analysis; twelve rats of them (four rats were randomly selected from control group, model group, and PRR group, respectively) were used for PCR array analysis. The remaining twelve of the fifty-two rats were also randomly divided into four groups (control, model, PRR, and UDCA groups) and only used for quantitative Real-Time PCR (qRT-PCR) analysis and acylcarnitine detection to validate the effects of PRR on fatty acid β-oxidation. All animals were maintained for 1 week at standard temperature (23 ± 2°C) and humidity (60 ± 5%) in a controlled room with a light/dark cycle of 12 h/12 h. During this period, the animals were allowed free access to food and water. Before experiments, the rats fasted for 12 h with free access to water. All experimental procedures were approved by the Biomedical Ethical Committee of Peking University (LA2015134) and conducted in accordance with the Guide for Care and Use of Laboratory Animals, published by the US National Institutes of Health (revised 2010).

It was reported that PRR could significantly alleviate cholestasis of model rats induced by ANIT with no toxic effect at the doses of 1, 9, 18, 36, 54, 72, and 81 g/(kg·d) PRR extract (equivalent to 2.86, 25.75, 51.50, 103, 154.50, 206, and 231.76 g/(kg·d) crude drug) in a dose-dependent manner (Wei et al., 2011; Ma et al., 2015). Based on these, the dosage adopted in this study was 20 g/kg PRR extract (equivalent to 55.48 g/kg crude drug) twice a day.

**FIGURE 1:**
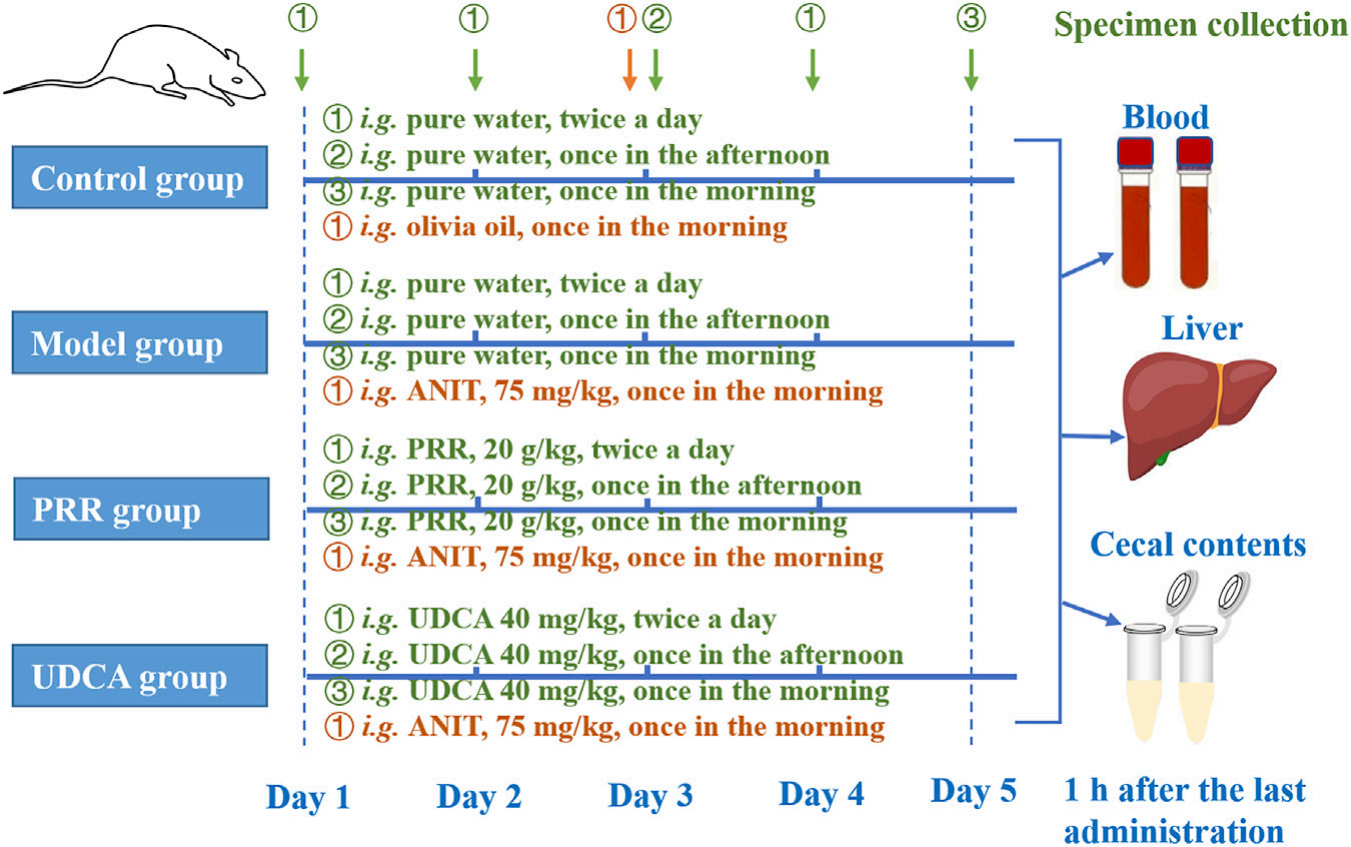
The dosage regimens of each group in this study. *i. g.*: intragastrical administration; PRR, Paeoniae Radix Rubra; UDCA, ursodeoxycholic acid; ANIT, α-naphthyl isothiocyanate.

Figure 1 shows the detailed dosage regimens of each group. The rats in the PRR group were intragastrically treated with 20 g/kg PRR extract (dissolved in pure water) twice per day for 4 days. Correspondingly, rats in the UDCA group were intragastrically treated with 40 mg/kg UCDA (the positive control, dissolved in pure water) twice per day for 4 days. Meanwhile, rats in model, PRR, and UDCA group were intragastrically treated with 75 mg/kg ANIT (dissolved in olive oil) to induce cholestasis in the third morning. The doses of UDCA and ANIT adopted in this study was based on relevant report (Wang et al., 2017).

### 2.4 Sample collection and preparation

One hour after the last administration of PRR extract, the rats (used for pharmacodynamic evaluation, targeted metabonomics analysis, gut microbiota analysis, and PCR array analysis) were anesthetized and blood samples were collected and centrifuged at 3,500 r/min for 15 min to separate serum. The serum samples thus obtained were stored at −80°C before determining the biochemical indexes and metabolites. Then the liver tissue was immediately removed and divided into two subsets. For the histological examination, the same lobe of liver tissue from each group was fixed in 10% (v/v) formalin for at least 24 h. To determine the mRNA expression levels, 20 mg of liver tissue was added in 1 ml TRIzol reagent before storage at −80°C. In addition, cecal contents for gut microbiota analysis were collected into sterile centrifuge tubes and snap-frozen in liquid nitrogen and stored at −80°C until analysis.

For the rats that were used for the qRT-PCR analysis and acylcarnitine detection, 1 hour after the last administration of PRR extract, blood samples were collected and centrifuged at 3,500 r/min for 15 min to separate serum and the serum samples were stored at −80°C before determining the biochemical indexes (Supplementary Table S4). Except for three rats in the UDCA group, the liver tissues of the remaining nine rats in control group, model group, and PRR group were removed and divided into two subsets. For the qRT-PCR analysis, 20 mg of liver tissue was added in 1 ml TRIzol reagent before storage at −80°C. To detect the acylcarnitines, 10 mg of liver tissue was collected and snap-frozen in liquid nitrogen and stored at −80°C until analysis.

### 2.5 Biochemical analysis

The serum biochemical indexes, including alkaline phosphatase (ALP), aspartate aminotransferase (AST), alanine aminotransferase (ALT), total bile acid (TBA), total bilirubin (TBIL), direct bilirubin (DBIL), and γ-glutamyl transpeptadase (GGT) were determined using standard routine procedures on a Beckman CX5 automatic biochemical analyzer (Beckman Coulter, Inc., United States) in the clinical laboratory of Peking University Third Hospital and on a Hitachi 7170 automatic biochemical analyzer (Hitachi, Ltd., Japan) in the Peking University Health Science Center Department of Laboratory Animal Science.

### 2.6 Histological examination

The fixed liver tissue was dehydrated in alcohol, embedded in paraffin, processed to 3–5 μm slices, stained with hematoxylin and eosin (H&E), and observed under a light microscope (Olympus Corporation, OLYMPUS CX21). Photographs of each slide were taken at ×200 magnification.

### 2.7 Determination of endogenous metabolites

The levels of fatty acids, amino acids, bile acids, organic acids, and acylcarnitines were determined with the general and validated procedures of analysis and testing platform of Peking University. Details are provided in Supplementary Information S4.

### 2.8 Detection of gut microbial community

Total genomic DNA from cecal content samples was extracted using the CTAB/SDS method (Osena et al., 2017). The 16S rRNA gene comprising V3–V4 regions was amplified by PCR using universal bacterial primers which were 341F (5′-ACT​CCT​ACG​GGA​GGC​AGC​AG-3′) and 806R (5′-GGACTACHVGGGTWTCTAAT-3′). The conditions were as follows: 98°C for 3 min followed by 30 cycles of 98°C for 10 s, annealing at 50°C for 30 s, and elongation at 72°C for 30 s, then followed by a final elongation at 72°C for 5 min. After PCR amplification, sequencing was performed on an Illumina Novaseq 6,000 platform by Beijing igeneCode Biotech Co., Ltd. (Beijing, China).

Raw data were demultiplexed and quality filtered according to the methods of Fadrosh et al. (2014). Cleaned tags were obtained by Fast Length Adjustment of Short reads (FLASH, v1.2.11) (Magoc and Salzberg, 2011). Operational taxonomic units (OTUs) were cleaned tags which were clustered at 97% similarity by UPARSE (v7.0.1090) (Edgar, 2013). The rarefaction curves and Shannon indexes were performed to evaluate the α diversity, which are used to explain the species richness and evenness of samples within one group, by using the software of mothur (v1.31.2) (http://www.mothur.org/wiki/Calculators). OTU rank curve and linear discriminant analysis (LDA) were performed with R statistical package (v3.1.1). For β diversity analysis, which can reflect differences in the composition and structure of microbiota of samples between groups, clustering heat map based on Bray-Curtis diversity distance was performed with QIIME (v1.80).

### 2.9 PCR array analysis

To profile the changes in mRNA expression related to fatty acids, the RT^2^ Profiler PCR Array Kit (Qiagen, Hilden, Germany) was used. This commercial array includes 84 preselected genes involved in the enzymatic pathways of fatty acids (PARN-007ZA). Total RNA was extracted from livers using TRIzol. Single-stranded cDNA was synthesized from 1.5 μg of total RNA using an RT^2^ First Strand Kit (Qiagen) following the manufacturer’s protocols. The analysis was conducted using 2X SuperArray PCR master mix (Arraystar, Rockville, MD, United States) in a ViiA 7 Real-Time PCR System (APPlied Biosystems, Foster City, CA, United States). Thermal cycling conditions were as follows: 10 min at 95 °C, followed by 40 cycles of 95°C for 15 s and 60°C for 60 s. Fold changes were calculated using the ^ΔΔ^Ct method with the software provided by the manufacturer (Qiagen). Ct values >35 were considered as no expression. The five housekeeping genes (β2M, LDHA, HPRT1, RPLP1, and ACTB) included in the array were used as reference genes. All five housekeeping genes and their average Ct values did not greatly change. In accordance with the manufacturer’s instructions, the average Ct value of all housekeeping genes was used for normalization.

### 2.10 Quantitative RT-qPCR analysis

The RT-qPCR was applied to validate the mRNA expression of Cpt1a, Hadha, Ppara, Slc25a20, and Ehhadh in liver. Briefly, we extracted the total RNA using TRizol Reagent (Sigma, St. Louis, MO, United States). Two micrograms of the total RNA were reverse transcribed using a High-Capacity cDNA Reverse Transcription Kit (Thermo Fisher Scientific Inc., Waltham, MA, United States). RT-qPCR was conducted using 2X PCR master mix (Arraystar, Rockville, MD, United States) in a ViiA 7 Real-Time PCR System (APPlied Biosystems, Foster City, CA, United States). The primer sequences are shown in Supplementary Table S5. Target mRNA levels were normalized to those of GAPDH mRNA and expressed as fold change relative to the control group. Thermal cycling conditions were as follows: 10 min at 95°C, followed by 40 cycles of 95°C for 10 s and 60°C for 60 s.

### 2.11 Statistical analysis

All parameters were expressed as mean ± SD in each group. One-way analysis of variance (ANOVA) was used to compare multiple groups by using SPSS 20.0 software (SPSS Inc., Chicago, IL, United States). The difference between groups was considered to be statistically significant when *p < 0.05*. Principal component analysis (PCA), partial least squares-discriminate analysis (PLS-DA), and hierarchical cluster analysis were employed to compare the metabolite profiles of different groups by using SIMCA-P software (version 14.0, Umetrics Umea, Sweden) and GraphPad Prism 8.3 (GraphPad Software, Inc., San Diego, CA, United States). The correlation heat maps of metabolite-to-metabolite and metabolite-to-gut microbes genus were plotted based on the Spearman correlation coefficients (www.omicshare.com). Ingenuity pathway analysis was performed using metabonomics pathway analysis (MetPA) (http://www.metaboanalyst.ca).

## 3 Results

### 3.1 Pharmacodynamic effects of PRR on the ANIT-induced cholestatic model rats

#### 3.1.1 Effects of PRR on biochemical indexes

Biochemical analysis revealed that ANIT dramatically increased serum ALT and AST levels (*p < 0.01*), which indicates hepatic cell damage, as well as dramatically increased ALP, GGT, TBIL, DBIL, and TBA (*p < 0.01*), which are markers of intrahepatic cholestasis (Table 1, 2). Conversely, all the above biochemical indexes were significantly reduced (*p < 0.01* or *p < 0.05*) when rats were treated with PRR. The effects of UDCA on reducing these indexes were slightly stronger than those of PRR (Table 1, 2). These results suggested that the cholestatic rat model was successfully established and PRR could effectively alleviate ANIT induced cholestasis.

**TABLE 1:** Effects of PRR on serum ALT, AST, ALP, and GGT in ANIT-induced cholestatic model rats (*n* = 10).

Group	ALT (U/L)	AST (U/L)	ALP (U/L)	GGT (U/L)
**Control**	52.20 ± 3.39	94.90 ± 2.92	268.30 ± 19.60	0.80 ± 0.42
**Model**	1063.40 ± 151.04##	1704.60 ± 392.74##	937.30 ± 37.88##	7.90 ± 0.74##
**PRR**	346.30 ± 38.09**	655.60 ± 59.13**	763.10 ± 32.20**	5.70 ± 0.82*
**UDCA**	330.70 ± 28.33**	540.30 ± 68.78**	720.60 ± 89.05**	4.10 ± 1.37**

^##^ *p*< 0.01 *versus* control group. *p< 0.05, **p< 0.01 *versus* model group.

**TABLE 2:** Effects of PRR on serum TBIL, DBIL, and TBA in ANIT-induced cholestatic model rats (*n* = 10).

Group	TBIL (μmol/L)	DBIL (μmol/L)	TBA (μmol/L)
Control	1.09 ± 0.09	0.25 ± 0.08	11.54 ± 1.25
Model	126.25 ± 22.75^##^	109.81 ± 19.86^##^	420.83 ± 31.40^##^
PRR	56.90 ± 4.82^**^	44.40 ± 4.33^**^	292.50 ± 15.04^**^
UDCA	39.66 ± 12.75^**^	26.92 ± 7.51^**^	202.36 ± 39.46^**^

^##^ *p*< 0.01 *versus* control group. ^**^ *p*< 0.01 *versus* model group.

#### 3.1.2 Effects of PRR on histological changes in the liver tissue of ANIT-induced cholestatic model rats

**FIGURE 2:**
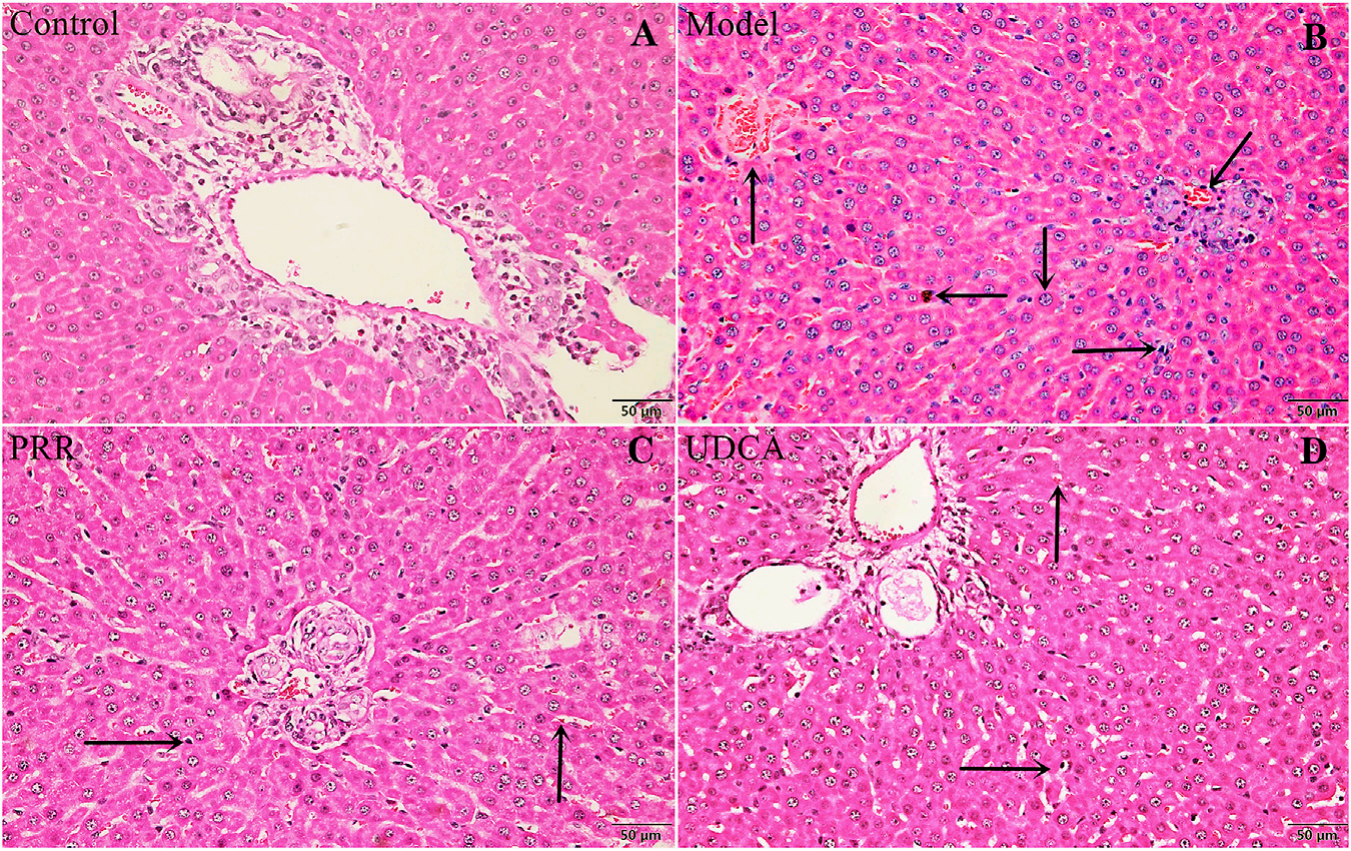
Effects of PRR on histological changes in the liver tissue of ANIT-induced cholestatic model rats. **(A)** Control group. **(B)** Model group. **(C)** PRR group. **(D)** UDCA group. Hepatocyte damage is indicated by black arrows: ← bile particles; ↑ sinusoid congestion; → infiltration with polymorphonuclear neutrophils; ↓ hepatocyte edema; ↙disordered hepatocyte cords. (H&E stained, ×200 magnification).

Histopathology results were in a good agreement with serum biochemistry results. As shown in Figure 2A, the hepatic tissues in control group exhibited normal cellular structures with intact hepatic lobules, liver cell cord in order, unexpanded hepatic sinuses, and no evidence of neutrophilic granulocyte infiltration.

In contrast, the ANIT-induced model group presented typical pathological changes including hepatocyte edema, disordered hepatocyte cords, serious sinusoid congestion, and infiltration with polymorphonuclear neutrophils (Figure 2B). Concurrently, the specimens of PRR and UDCA groups only exhibited mild sinusoid congestion and less neutrophil infiltration (Figures 2C,D).

### 3.2 Metabolic responses of ANIT-induced cholestatic model rats to PRR treatment

#### 3.2.1 Influence of PRR treatment on the metabolic phenotype

**FIGURE 3:**
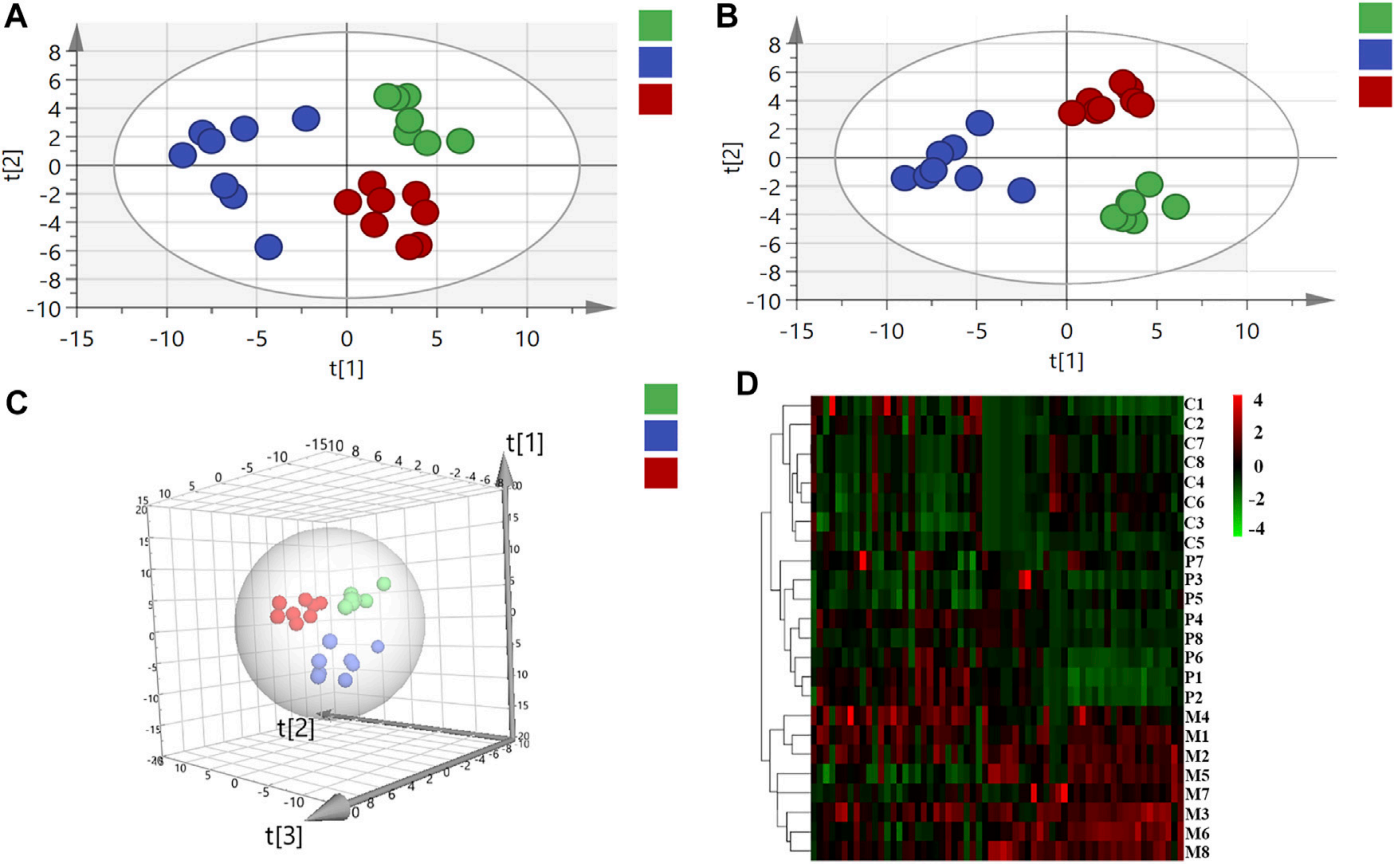
The relationship of metabolic phenotypes among three groups. **(A)** PCA plot. **(B)** PLS-DA plot. **(C)** 3D PLS-DA plot. **(D)** Hierarchical clustering heat map. In panel **(A–C)**, the green circles represent rats in the control group; the blue circles represent rats in the model group; red circles represent rats in the PRR group. In panel **(D)**, each row represents one sample, and each square represents a metabolite. The metabolites with higher levels than the average level of all metabolites in the sample are shown in red; the metabolites with lower levels than the average level of all metabolites in the sample are shown in green. The deeper color means the larger deviation from the average level of all metabolites in the sample. C1–C8 are the numbering of rats in control group; M1–M8 are the numbering of rats in model group; P1–P8 are the numbering of rats in PRR group.

In virtue of the important roles of fatty acids, bile acids, amino acids, and organic acids in the ANIT-induced cholestasis, to elucidate the mechanisms of PRR in the treatment of cholestasis, we first applied targeted metabonomics to analyze these types of metabolites in serum of rats in control group, model group, and PRR group. Totally, 75 metabolites were detected in the serum of rats (including 28 amino acids, 15 bile acids, 19 fatty acids, and 13 organic acids). Then several multivariate statistical analysis methods were combined to explore whether PRR could affect the above metabolites and its regulatory effects on them. Figures 3A–C show the score plots obtained by the unsupervised PCA and supervised PLS-DA. In these score plots, the metabolites and their concentrations determined the relative positions of the samples. Therefore, the relative positions of the plots suggested the differences among the samples. Obviously, the control group, model group, and PRR group were separated; and the PRR group was located much closer to the control group than the model group. The hierarchical clustering further visually displayed the similarity of metabolic phenotypes between PRR group and control group. In the hierarchical clustering trees, PRR group was finally clustered into a large group with control group (Figure 3D). The above results indicated that the ANIT-induced metabolic disorders were significantly alleviated with PRR treatment.

#### 3.2.2 Analysis on differential metabolites

From the 75 metabolites detected in the serum of rats, we found that rats intragastrically treated with ANIT exhibited a significant increase in the levels of 15 fatty acids, 11 bile acids (including 6 primary bile acids and 5 secondary bile acids), and 13 amino acids, as well as a significant decrease in the levels of 1 amino acid and 2 organic acids. Of these, treatment with PRR conspicuously reduced 15 fatty acids, 5 secondary bile acids, and 8 amino acids, and increased 2 organic acids to normal levels (Table 3).

**TABLE 3:** Significant changes in metabolites and their change trends between the model group and control group or between PRR group and model group.

No.	Metabolites	Categories	Model group/Control group	PRR group/Model group
1	FFA 14:0^a^	Fatty acids	↑**	↓**
2	FFA 20:1^a^		↑**	↓**
3	FFA 20:2^a^		↑**	↓**
4	FFA 20:3^a^		↑**	↓**
5	FFA 22:4^a^		↑**	↓**
6	FFA 22:5^a^		↑**	↓**
7	FFA 22:6^a^		↑**	↓**
8	FFA 18:0^a^		↑**	↓**
9	FFA 20:4^a^		↑**	↓**
10	FFA 16:0^a^		↑**	↓**
11	FFA 18:1^a^		↑**	↓**
12	FFA 18:2^a^		↑**	↓**
13	FFA 17:0^a^		↑**	↓**
14	FFA 17:1^a^		↑**	↓**
15	FFA 16:2^a^		↑**	↓**
16	Pyruvic acid	Organic acids	↓**	↑**
17	Citric acid		↓**	↑**
18	Taurohyodeoxycholic acid^b^	Bile acids	↑**	↓**
19	Tauroursodeoxycholic acid^b^		↑**	↓**
20	Taurolithocholic acid^b^		↑**	↓*
21	Glycoursodeoxycholic acid^b^		↑*	↓*
22	Deoxycholic acid^b^		↑**	↓**
23	Taurodeoxycholic acid^c^		↑**	↓
24	Cholic acid^c^		↑**	↓
25	Glycocholic acid^c^		↑**	↓
26	Glycochenodeoxycholic acid^c^		↑*	↓
27	Taurocholic acid^c^		↑**	↓
28	Taurochenodeoxycholic acid^c^		↑**	↓
29	L-Leucine	Amino acids	↑**	↓**
30	Isoleucine		↑**	↓**
31	β-Aminobenzoic acid		↑**	↓**
32	γ-Aminobenzoic acid		↑*	↓**
33	β-Alanine		↑**	↓**
34	L-Proline		↑**	↓**
35	L-Valine		↑**	↓**
36	L-Arginine		↑**	↓**
37	L-Sarcosine		↓**	↑
38	L-Glutamine		↑*	↓
39	L-Methionine		↑**	↓
40	L-Threonine		↑**	↓
41	Taurine		↑**	↓
42	L-Tyrosine		↑*	↓

*p< 0.05; ^**^ *p< 0.01*.

^a^ for the name of fatty acids, the number before the colon represents the number of carbon atoms; the number behind the colon represents the number of double bonds.

^b^ represents secondary bile acid.

^c^ represents primary bile acid.

Based on the above results, the number of abnormal fatty acids, bile acids, amino acids, and organic acids induced by ANIT accounted for 79% (15/19), 73% (11/15), 46% (13/28), and 15% (2/13), respectively, of the total number of metabolites of the corresponding type detected in the serum of rats. Meanwhile, the number of fatty acids, bile acids, amino acids, and organic acids reversed by PRR accounted for 100% (15/15), 45% (5/11), 62% (8/13), and 100% (2/2), respectively, of the number of abnormal metabolites of the corresponding type (Table 4). Therefore, the proportion of fatty acids both disturbed by ANIT and reversed by PRR was the highest.

**TABLE 4:** Effects of ANIT and PRR on the four types of metabolites.

Types of metabolites	Number of metabolites detected	Number of abnormal metabolites induced by ANIT	Number of metabolites regulated by PRR
Fatty acids	19	15	15
Bile acids	15	11	5
Amino acids	28	13	8
Organic acids	13	2	2

**FIGURE 4:**
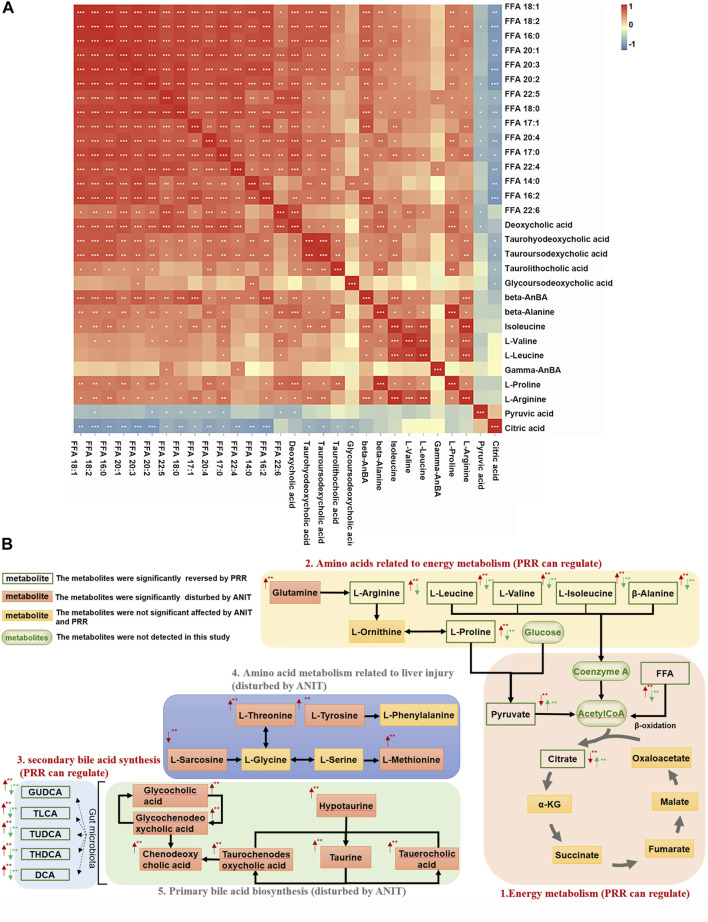
Associations among metabolites reversed by PRR. **(A)** Correlation analysis between metabolites reversed by PRR. **(B)** The metabolic network of metabolites disturbed by ANIT and reversed by PRR. In panel **(A)**, the blue color denotes that there is negative correlation between the metabolite in the row and the metabolite in the column (the correlation coefficient is less than 0); the red color denotes that there is positive correlation between the metabolite in the row and the metabolite in the column (the correlation coefficient is greater than 0); *p < 0.05; **p < 0.01; ***p < 0.001. In panel **(B)**, the red arrow represents the change in trend of the model group compared with the control group. The green arrow represents the change in trend of the PRR group compared with the model group. *p < 0.05; **p < 0.01.

Then metabolite-to-metabolite correlation was revealed by Spearman correlation analysis (Figure 4A). There were general positive correlations between 15 fatty acids and 5 secondary bile acids, between 15 fatty acids and 8 amino acids, and between 5 secondary bile acids and 8 amino acids (correlation coefficient>0, *p < 0.05* or *p < 0.01*). Meanwhile, there were negative correlations between 15 fatty acids and 2 organic acids, as well as between 5 secondary bile acids and 2 organic acids (correlation coefficient<0, *p < 0.05* or *p < 0.01*). However, there were no general correlations observed between 2 organic acids and 8 amino acids.

#### 3.2.3 Analysis of metabolic pathways

Ingenuity pathway analysis was implemented with MetPA to appraise the metabolic pathways affected by PRR treatment. The results showed that ANIT induced the dysregulation of 15 pathways through 16 metabolites (β-alanine, L-arginine, etc.), whereas we found that PRR could significantly alleviate 8 pathways of them through 6 metabolites (β-alanine, L-arginine, etc.) (Table 5).

**TABLE 5:** Pathways and related metabolites significantly affected by ANIT or PRR.

Pathway	Model group		PRR group	
	The related metabolites	Impact	The related metabolites	Impact
β-alanine metabolism	β-alanine	0.40	β-alanine	0.40
Pyruvate metabolism	Pyruvic acid	0.21	Pyruvic acid	0.21
Citrate cycle (TCA cycle)	Pyruvic acid, citric acid	0.14	Pyruvic acid, citric acid	0.14
Arginine and proline metabolism	L-arginine, L-proline, pyruvic acid	0.14	L-arginine, L-proline, pyruvic acid	0.14
Glycolysisx/gluconeogenesis	Pyruvic acid	0.10	Pyruvic acid	0.10
Arginine biosynthesis	L-glutamine, L-arginine	0.076	L-arginine	0.076
Glyoxylate and dicarboxylate metabolism	Pyruvic acid, citric acid	0.032	Pyruvic acid, citric acid	0.032
Pantothenate and CoA biosynthesis	L-valine, β-alanine	0.021	L-valine, β-alanine	0.021
Phenylalanine, tyrosine, and tryptophan biosynthesis	L-tyrosine	0.5		
Taurine and hypotaurine metabolism	Taurine	0.43		
Tyrosine metabolism	L-tyrosine, pyruvic acid	0.14		
Primary bile acid biosynthesis	Taurine, taurocholic acid, glycochenodeoxycholic acid, glycocholic acid, taurochenodeoxycholic acid	0.11		
Alanine, aspartate, and glutamate metabolism	L-glutamine, citric acid, pyruvic acid	0.11		
*Glycine*, serine, and threonine metabolism	L-sarcosine, L-threonine, pyruvic acid	0.11		
Cysteine and methionine metabolism	L-methionine, pyruvic acid	0.10		

#### 3.2.4 Analysis of the metabolic network

**FIGURE 5:**
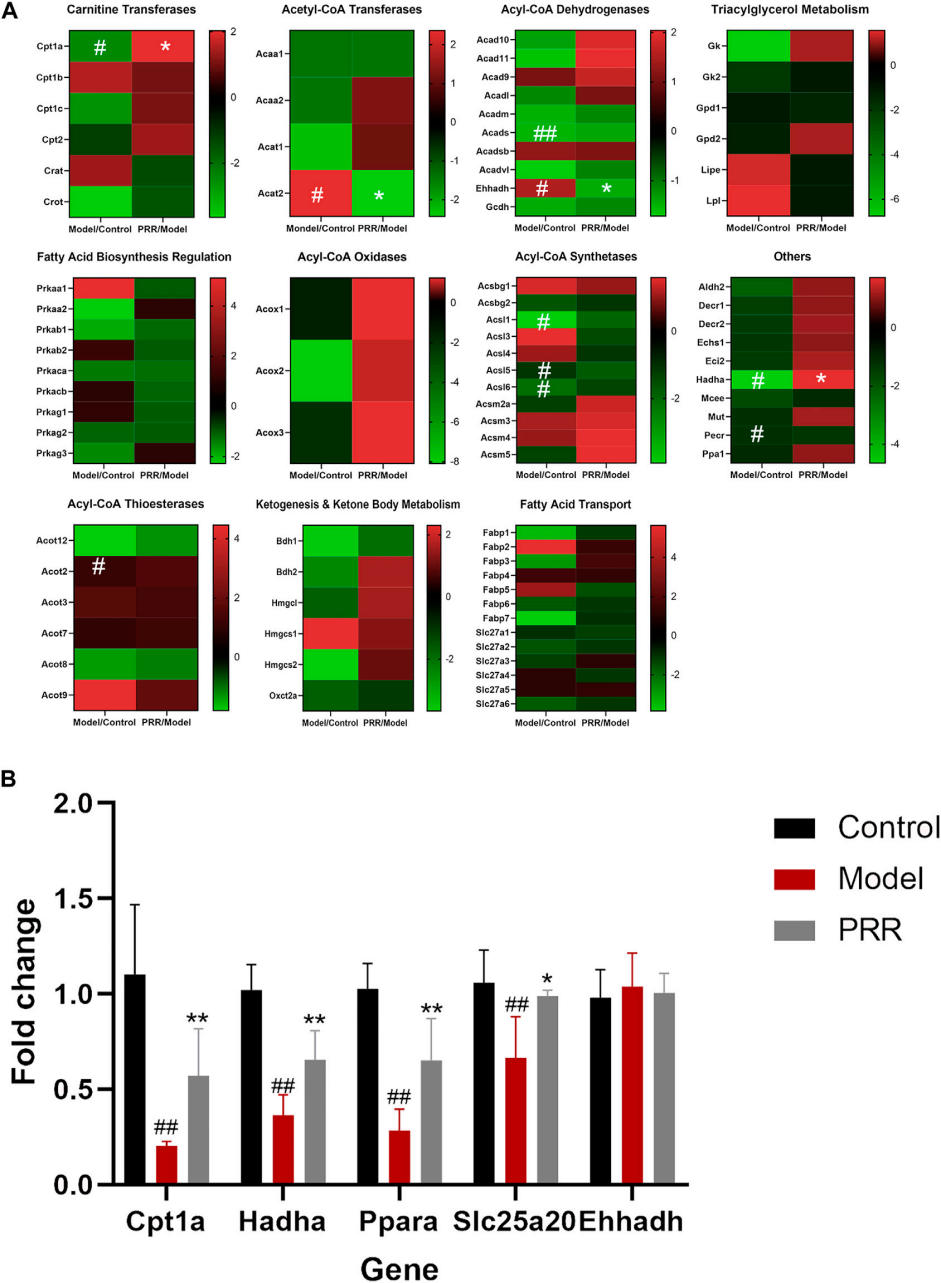
PRR modulates the expression of key fatty acid-related genes. **(A)** Heat map of PCR array results. **(B)** RT-qPCR validation of PCR array data. In panel (A), according to the type of enzyme, the expression of 84 genes is shown in different heat maps. In each heat map, the left column is the expression of the genes in the model group compared with the normal group; the right column shows the expression of the genes in the PRR group compared with the model group. Green represents downregulation; red represents upregulation. The deeper red or deeper green indicates that the gene is more significantly upregulated or downregulated. ^#^ *p* < 0.05, ^##^ *p* < 0.01 *versus* control group; **p < 0.01, *p < 0.05 *versus* model group.

Based on the pathway analysis, we found that there were certain links among 28 of the 30 metabolites (except β-aminobenzoic acid and γ-aminobenzoic acid) that were regulated by PRR (Figure 4B). Namely, 15 fatty acids (FFA 14:0, FFA 20:1, etc.) and 6 amino acids (L-arginine, β-alanine, etc.) (Figures 4B–1, 4B–2), which were downregulated by PRR, and 2 organic acids (pyruvic acid and citric acid) (Figures 4B–1), which were upregulated by PRR, were all associated with energy metabolism. Meanwhile, PRR also could downregulate 5 secondary bile acids (Figures 4B–3), which were important signal molecules in energy metabolism. However, for the 4 amino acids not related to energy metabolism and 6 primary bile acids, PRR exerted no significant regulatory effects (Figures 4B–4, 4B–5). Therefore, PRR could regulate metabolic disorders caused by ANIT with the focus on improving energy metabolism.

Considering the importance of fatty acids and secondary bile acids in energy metabolism, fatty acids and secondary bile acids were selected for further exploration.

### 3.3 The regulatory effects of PRR on the genes and intermediates related to fatty acids

#### 3.3.1 Identification of fatty acid-related genes that were significantly regulated by PRR

Targeted metabonomics was applied to analyze the fatty acids in liver tissues of rats in control group, model group, and PRR group. We found that nine fatty acids were significantly elevated, and PRR could reduce these nine fatty acids (Supplementary Table S6). These indicated that PRR could alleviate the accumulation of fatty acids in liver tissue. Then, to explore the mechanisms of PRR regulating fatty acid metabolism, we used a commercially available rat RT^2^ Profiler PCR array to profile the expression of 84 key fatty acid-related genes. Heat maps of PCR array data showed that ANIT could significantly inhibit the expression of Cpt1a, Acads, Acsl1, Acsl5, Acsl6, Hadha, and Pecr, and significantly enhance the expression of Acat2, Ehhadh, and Acot2 (Figure 5A). PRR could reverse the abnormal changes of Cpt1a, Acat2, Ehhadh, and Hadha (Figure 5A), among which Cpt1a, Ehhadh, and Hadha are fatty acid β-oxidation genes, indicating that fatty acid β-oxidation might play important role in the treatment of cholestasis by PRR.

#### 3.3.2 Verifying selected genes related to PRR treatment

To validate the regulatory effects of PRR on fatty acid β-oxidation genes, the expression of Cpt1a, Ehhadh, Hadha, Ppara, and Slc25a20 (Ppara and Slc25a20 are two fatty acid β-oxidation genes, but not contained in PCR array) were determined by RT-qPCR. The results showed that ANIT significantly inhibited the expression of Cpt1a, Hadha, Ppara, and Slc25a20 (Figure 5B). In contrast, treatment with PRR significantly enhanced the expression of Cpt1a, Hadha, Ppara, and Slc25a20. These results verified that PRR could enhance the expression of fatty acid β-oxidation genes to attenuate the impaired fatty acid β-oxidation induced by cholestasis.

#### 3.3.3 Influence of PRR treatment on acylcarnitines

**FIGURE 6:**
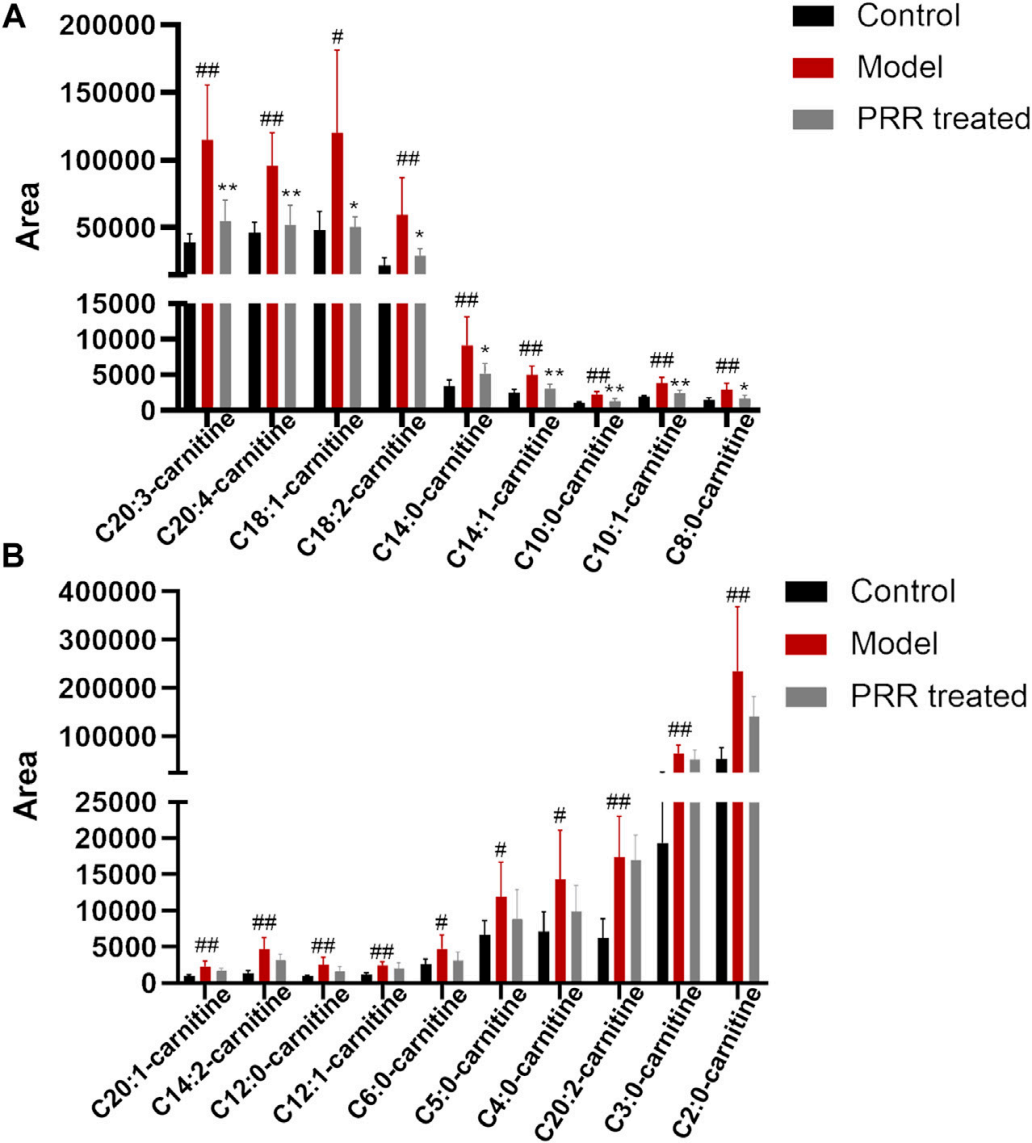
Changes in acylcarnitine levels among the three groups. **(A)** Abnormal acylcarnitines in the model group that can be significantly reversed by PRR treatment. **(B)** Abnormal acylcarnitines in the model group that cannot be significantly reversed by PRR treatment. ^#^ *p* < 0.05, ^##^ *p* < 0.01 *versus* control group. *p < 0.05, **p < 0.01 *versus* model group. For the name of acylcarnitines, the number before the colon represents the number of carbon atoms; the number behind the colon represents the number of double bonds.

Further, we investigated the influence of PRR on the level of acylcarnitines (the forms of fatty acids into mitochondria for β-oxidation). Among the 25 acylcarnitines in the liver identified by LC-MS, the levels of 19 acylcarnitines increased significantly in the model group, whereas PRR markedly reversed the levels of 9 acylcarnitines (Figure 6A). The levels of the other 10 acylcarnitines also could be reduced by PRR, but the differences were not significant (Figure 6B). These results demonstrated that fatty acids could not be normally transported into mitochondria in cholestatic rats, whereas PRR could promote the transportation of fatty acids into mitochondria for fatty acid β-oxidation. These results confirmed that PRR could effectively attenuate the impaired fatty acid β-oxidation induced by cholestasis.

### 3.4 Modulatory effects of PRR on the gut microbiota in cholestatic model rats

Given that secondary bile acids are formed by gut microbiota, to further elucidate the possible mechanisms of PRR in regulating secondary bile acids, we explored whether PRR could alleviate the gut microbiota disorders induced by cholestasis and the relationship between the changes in the gut microbiota and secondary bile acids *via* 16S rRNA gene sequencing analysis and Spearman’s correlation coefficient analysis.

#### 3.4.1 Diversity analysis of gut microbiota

**FIGURE 7:**
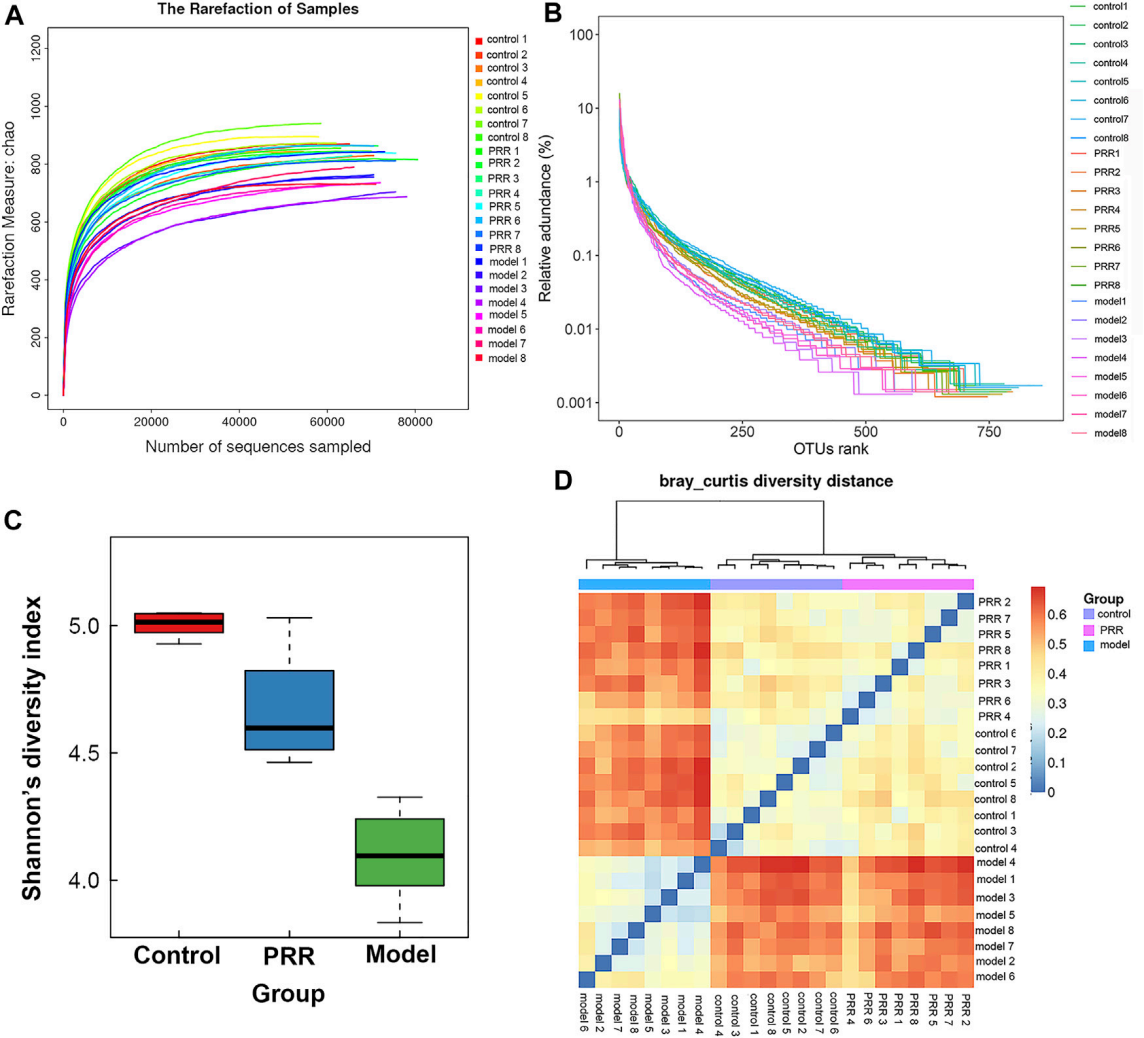
Diversity of microbiota among the PRR, control, and model groups. **(A)** Evaluation of the sequencing quality based on rarefaction analyses. **(B)** α diversity evaluation based on OTU rank curves. **(C)** α diversity evaluation based on Shannon’s diversity indexes (mean ± SD). **(D)** β diversity evaluation based on Bray-Curtis diversity distance. The color of square represents the distance between the sample in the row and the sample in the column.

The rarefaction curve can reflect the sequencing depth for sample. It was found that the rarefaction curves of all samples had a platform stage, demonstrating that the sequencing depth had covered rare new phenotypes and species to the greatest extent (Figure 7A). OTU rank curve and Shannon’s diversity index are the common microbial α diversity indexes, which are used to explain the species richness and evenness of samples within one group. The wider and smoother OTU rank curves and the larger Shannon’s diversity index indicates that the sample is with a higher α diversity. As shown in Figures 7B,C, ANIT resulted in narrower, steeper OTU rank curve, and a reduced Shannon’s diversity index. However, PRR intervention could make OTU rank curve wider and flatter and Shannon’s diversity index larger, suggesting that PRR significantly enriched the microbial α diversity. Then Bray-Curtis diversity distance analysis was conducted to evaluate β diversity, which can reflect the difference in the composition and structure of microbiota of samples among groups. In the clustering heat map based on Bray-Curtis diversity distance, the distance between samples is closer, indicating that there is little difference in the composition and structure of microbiota. The PRR group was much closer to the control group rather than the model group, and the PRR group was finally clustered into a large group with the control group (Figure 7D), indicating that PRR treatment could help to restore the microbiota dysfunction induced by ANIT.

#### 3.4.2 Analysis on gut microbiota composition

**FIGURE 8:**
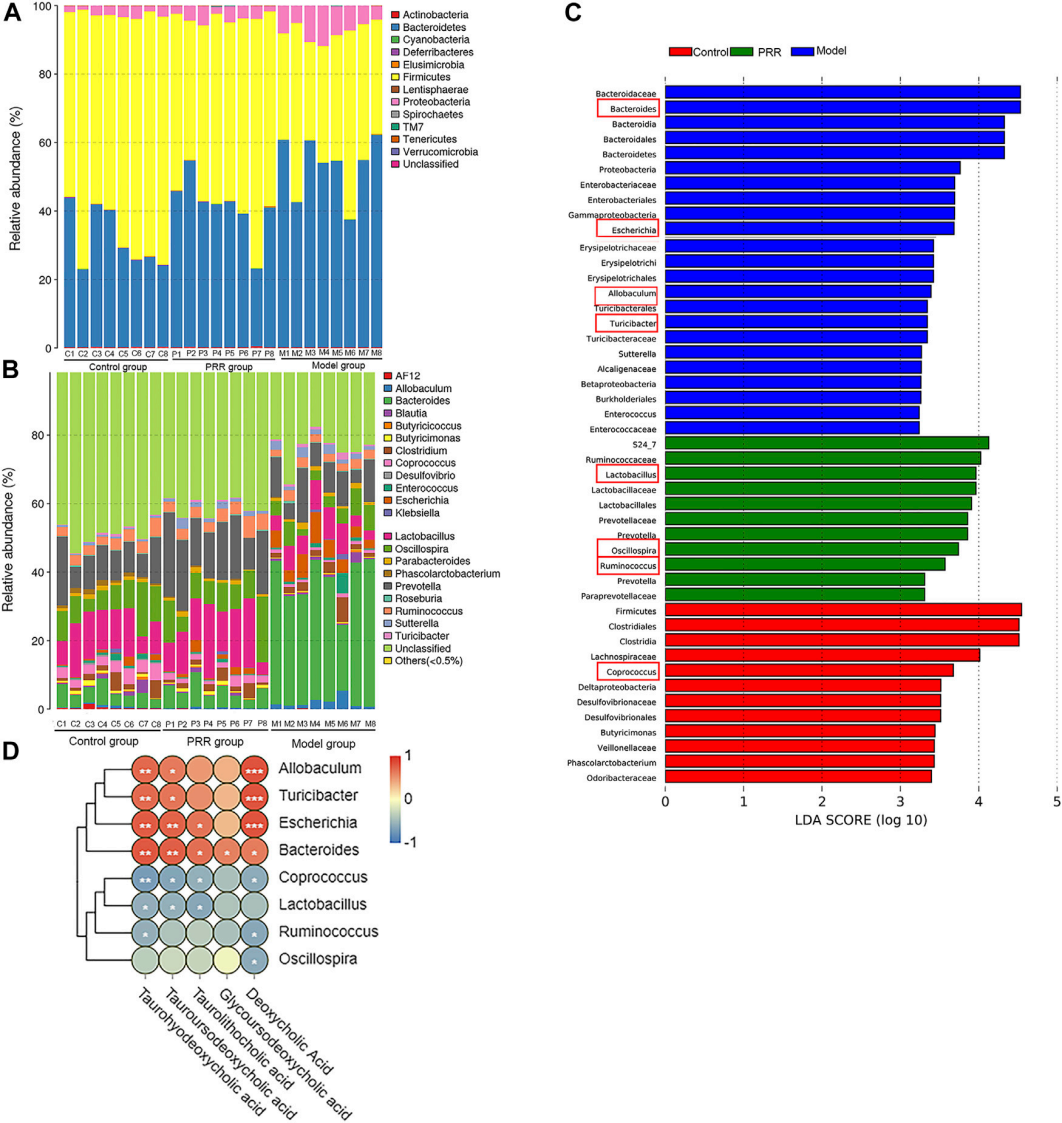
Regulation of PRR on gut microbiota composition. **(A)** Bacterial taxonomic profiling in the phylum level of gut microbiota. **(B)** Bacterial taxonomic profiling in the genus level of gut microbiota. **(C)** Linear discriminant analysis (LDA) scores of the discriminative taxa, showing the dominant genera of each group, among which the genera listed in the left and in red box are significantly regulated by PRR. **(D)** Correlation analysis between gut microbiota and secondary bile acids; blue denotes that there is negative correlation between the genera in the row and the secondary bile acid in the column (the correlation coefficient is less than 0); red denotes that there is positive correlation between the genera in the row and the secondary bile acid in the column (the correlation coefficient is greater than 0); *p < 0.05; **p < 0.01; ***p < 0.001; glycoursodeoxycholic acid is negative correlated to *Coprococcus*, *Lactobacillus*, *Ruminococcus*, and *Oscillospira* without significant differences.

We analyzed the gut microbiota composition in the control, model, and PRR groups. The relative abundance of phyla in each sample are showed in Figure 8A, which demonstrated that the model group had higher relative abundance of *Bacteroidetes* and *Proteobacteria* and lower relative abundance of *Firmicutes* than the control group. However, PRR treatment could remarkably reverse these changes. Further, there were more obvious differences in the microbial community profiles at the genus level among the three groups than that at the phylum level (Figure 8B). In addition, the genus is the lowest level that 16S rDNA could accurately identify, so we used Wilcoxon rank-sum test to find the gut microbiota which were significantly regulated by PRR at the genus level. The results showed that the relative abundance of 8 main genera (their relative abundance in each group >0.1) was significantly altered by ANIT treatment and was restored with PRR treatment (Table 6). Combined with the histogram of distribution of LDA values, PRR could significantly downregulate the relative abundance of *Bacteroides*, *Escherichia*, *Turicibacter*, and *Allobaculum* to normal, which were the dominant genera (Figure 8C) of model group. PRR could significantly upregulate their relative abundance to normal of three dominant genera (*Lactobacillus*, *Ruminococcus*, and *Oscillospira*) (Figure 8C) of the PRR group and one dominant genus (*Coprococcus*) (Figure 8C) of the control group. These results indicated that PRR could remarkably alleviate the gut microbiota disorders induced by ANIT.

**TABLE 6:** Relative abundance of eight major bacteria genera among three groups (n = 8).

Major genus	Relative abundance (%)			p-values		
	Control group	Model group	PRR group	Model vs. Control	PRR vs. Model	PRR vs. Control
*Bacteroides*	4.48 ± 1.80	35.95 ± 8.08	5.39 ± 2.42	<0.001	<0.001	0.44
Coprococcus	3.35 ± 0.96	0.77 ± 0.16	1.74 ± 0.72	<0.001	0.0011	<0.001
*Escherichia*	0.84 ± 0.89	4.91 ± 2.19	1.37 ± 1.12	<0.001	0.0010	0.28
*Lactobacillus*	11.03 ± 3.72	6.14 ± 2.66	13.29 ± 6.10	0.015	0.015	0.39
Ruminococcus	2.42 ± 0.76	1.01 ± 0.31	3.18 ± 1.01	<0.001	<0.001	0.28
Oscillospira	8.43 ± 3.58	4.89 ± 2.30	9.18 ± 4.63	0.021	0.028	0.96
Turicibacter	0.36 ± 0.25	0.87 ± 0.52	0.31 ± 0.20	0.010	<0.005	0.80
Allobaculum	0.40 ± 0.26	1.90 ± 1.61	0.27 ± 0.20	<0.001	<0.005	0.28

*p< 0.05* was considered statistically significant.

#### 3.4.3 Correlation analysis between gut microbiota and secondary bile acids

We next applied Spearman’s correlation coefficient analysis to investigate whether changes in the gut microbiota and secondary bile acids were correlated. The heat map reflected the relative abundance of the above eight genera were all associated with the levels of the five secondary bile acids downregulated by PRR (Figure 8D). Specifically, the altered secondary bile acids (downregulated by PRR) were positively correlated with *Bacteroides*, *Escherichia*, *Turicibacter*, and *Allobaculum* (downregulated by PRR), but negatively correlated with *Coprococcus*, *Lactobacillus*, *Ruminococcus*, and *Oscillospira* (upregulated by PRR).

## 4 Conclusions and discussion

Under the guidance of targeted metabonomics, this study clarified the mechanisms of PRR treating cholestasis by PCR array and 16S rRNA analyses. As the result, we found that PRR could significantly reverse the elevation of fatty acids (FFA 14:0 and other 14 fatty acids) and the decrease of organic acids (pyruvic acid and citric acid) in the serum of cholestatic model rats. Meanwhile, 8 elevated amino acids (L-proline, β-alanine, etc.) and 5 elevated secondary bile acids (taurohyodeoxycholic acid, tauroursodeoxycholic acid, etc.), which can be reduced by PRR, are also reported herein for the first time. Further exploration revealed for the first time that PRR could enhance fatty acid β-oxidation and alleviate gut microbiota disorders, which may be the way for PRR to reduce 15 fatty acids and 5 secondary bile acids in the serum of cholestatic model rats. This study provides new insights into the anti-cholestasis mechanisms of PRR from the aspects of host metabolism and gut microbiota. Given the findings about the regulatory effects of PRR on the gut microbiota and bile acids in this work, we will carry out some researches to further elucidate the mechanisms of PRR in the treatment of cholestasis from gut microbiota-bile acids-FXR/TGR5 in the future.

### 4.1 The fatty acids, amino acids, organic acids, and secondary bile acids regulated by PRR were associated with energy metabolism

In the present study, ANIT significantly elevated the levels of 15 fatty acids, 13 amino acids, 11 bile acids (6 primary bile acids and 5 secondary bile acids), and reduced the levels of 1 amino acid (L-sarcosine) and 2 organic acids, which were consistent with the existing literature (Lin et al., 2019; Liu et al., 2021; Waters et al., 2001). Meanwhile, we found that of these, PRR could remarkably reverse the elevation of 15 fatty acids (FFA 14:0, FFA 20:1, etc.), 8 amino acids (L-arginine, β-alanine, etc.), 5 secondary bile acids (taurohyodeoxycholic acid, tauroursodeoxycholic acid, etc.), and the decrease of 2 organic acids (pyruvic acid and citric acid) (Table 3). The increase in fatty acid levels and the decrease in organic acids levels reversed by PRR is reported for the first time by this study. Ma et al. reported that PRR could significantly upregulate 3 metabolites and downregulate 5 metabolites in the serum of ANIT-induced cholestatic model rats (Ma et al., 2016). However, there were no fatty acids or organic acids specified in their study, and the amino acids and bile acids that Ma et al. mentioned only included L-palmitoylcarnitine, 4-guanidinobutanoate, pantothenate, D-arginine, 2-phenylacetamide, glycocholic acid, glycochendeoxycholic acid, and taurocholic acid. Therefore, the 8 elevated amino acids and 5 elevated secondary bile acids in cholestatic rats, which can be reversed by PRR (Table 3) in our research, are also found for the first time.

#### 4.1.1 Fatty acids, amino acids, and organic acids regulated by PRR were related to the TCA cycle

Fatty acids are important substrates in energy metabolism, which can be catabolized to acetyl coenzyme A and enter the TCA cycle; branched chain amino acids (L-leucine, L-valine, etc.), β-alanine (Luo et al., 2021), L-proline, and L-arginine (Tanner et al., 2018) can also be catabolized to acetyl coenzyme A or α-ketoglutaric acid to participate in the TCA cycle. Meanwhile, pyruvic acid and citric acid are important intermediates of the TCA cycle. In this study, ANIT increased 15 fatty acids and 6 amino acids (β-alanine, L-proline, L-arginine, L-leucine, isoleucine, and L-valine) and reduced 2 organic acids (pyruvic acid and citric acid) (Table 3). Therefore, we considered that ANIT might inhibit these 15 fatty acids and 6 amino acids to enter the TCA cycle to induce the dysfunction of energy metabolism (manifested as a decrease of the levels of pyruvic acid and citric acid). Further, PRR reversed the abnormal changes of these 15 fatty acids, 6 amino acids, and 2 organic acids (Table 3); thus, we also considered that PRR could promote these fatty acids and amino acids entering the TCA cycle to alleviate the energy metabolism disorder (manifested as an increase of the levels of pyruvic acid and citric acid). However, 4 amino acids involving in 6 amino acid metabolic pathways (Table 5), which contributed to the liver function impairment (Table 7), could not be alleviated by PRR.

**TABLE 7:** The functions of amino acid pathways affected by ANIT or PRR.

Pathways	Changes induced by ANIT	Function in the pathogenesis of cholestasis	Reversed by PRR
β-alanine metabolism	β-alanine can be catabolized to coenzyme A to participate in fatty acid oxidation, amino acid decomposition, and the TCA cycle, thereby regulating 90% of energy metabolism Luo et al. (2021). Thus, the increase of β-alanine in serum of model rats may reduce the production of coenzyme A, resulting in energy supply dysfunction	Aggravate energy metabolism disorder	Yes
Pantothenate and CoA biosynthesis			
Arginine and proline metabolism	L-proline and L-arginine can be oxidized to P5C and then catabolized to coenzyme A or α-ketoglutaric acid to participate in TCA cycle Tanner et al. (2018). Therefore, the increase of L-proline in serum of model rats may cause energy supply dysfunction		
Arginine biosynthesis			
Phenylalanine, tyrosine, and tryptophan biosynthesis	L-tyrosine can be metabolized to toxic intermediate metabolites such as fumarate acetate, maleyl acetate, and succinyl acetone, which can damage hepatocytes by inducing an endoplasmic reticulum stress response, activating the Akt/MEK pathway and the NRF2 transcription factor Tanguay et al. (2017). Hence, the increase of L-tyrosine in serum of model rats may lead to the accumulation of these toxic metabolites, resulting in liver injury	Impair liver function	No
Tyrosine metabolism			
Alanine, aspartate, and glutamate metabolism	The disturbance of this metabolic pathway can generate a vicious circle between the synthesis and decomposition of glutamate, resulting in an increase of blood ammonia, which can damage hepatocytes by initiating mitochondrial apoptosis Holecek (2014). Thus, the remarkably elevated L-glutamine in serum of model rats may cause liver injury by increasing blood ammonia		
*Glycine*, serine, and threonine metabolism	The abnormal levels of L-threonine can increase ammonia and uric acid levels in the blood and the activities of amino acid oxidase and urea synthase in the liver to accelerate the degradation of protein Kidd and Kerr (1996). Therefore, a significant increased L-threonine in serum of model rats may lead to amino acid imbalance and induce liver injury		
Cysteine and methionine metabolism	In this pathway, L-methionine can be converted to S-adenosylmethionine which is essential for the function of different metabolic pathways through transmethylation. The reduced utilization of SAM can lead to various liver diseases Pascale et al. (2019). Therefore, the increased L-methionine in serum of model rats may cause liver injury by inhibiting the utilization of SAM.		
Taurine and hypotaurine metabolism	Taurine has a variety of physiological functions, including promoting bile acid excretion, antioxidation, and antihepatotoxicity Miyazaki and Matsuzaki (2014). Thus, the increase of taurine in serum of model rats can protect the damaged liver to a certain extent		

#### 4.1.2 PRR can regulate five secondary bile acids which are important signaling molecules

Secondary bile acids are important signaling molecules, which can maintain the balance of energy metabolism through activating receptors including takeda G-protein-coupled receptor 5 (TGR5) and farnesoid X receptor (FXR). (Hylemon et al., 2009). For example, Watanabe et al. found that secondary bile acids (tauroursodeoxycholic acid, taurolithocholic acid, etc.) could activate TGR5, resulting in the increased expression of type 2 deiodinase and the elevation of triiodothyronine; triiodothyronine induced uncoupling protein expression to eliminate proton gradient difference in electron transport chain and inhibit ATP synthesis (Watanabe et al., 2006). Several reports found that tauroursodeoxycholic acid, taurolithocholic acid, and deoxycholic acid were agonists of TGR5, and taurohyodeoxycholic acid and glycoursodeoxycholic acid could act on FXR (Poland and Flynn, 2021). Thus, we considered that the downregulation of these five secondary bile acids (taurohyodeoxycholic acid, tauroursodeoxycholic acid, etc.) by PRR observed in this study could contribute to the alleviation of energy metabolism disorders.

Since secondary bile acids not only can cause cell death or may cause senescence, but also can generate or maintain a chemokine and cytokine response, accumulation of secondary bile acids is currently considered as a driving force of cholestatic liver injury (Cai and Boyer, 2021). Therefore, the observation that PRR reduced secondary bile acids in this study indicates that PRR can contribute to the alleviation of liver injury.

### 4.2 The enhancement of PRR on fatty acid β-oxidation may be the way to reduce the serum fatty acids levels

Fatty acid β-oxidation in mitochondria is essential for maintaining energy homeostasis. In fatty acid β-oxidation, fatty acids are first activated to acyl coenzyme A, then acyl coenzyme A is transported into mitochondria, and finally degraded to acetyl coenzyme A by oxidation, hydration, a second oxidation, and thiolysis. A series of genes (Cpt1a, Cpt2, Hadha, etc.) encoding corresponding enzymes are involved in fatty acid β-oxidation (Houten et al., 2016). Our study found that ANIT could inhibit the expression of four fatty acid β-oxidation related genes (Cpt1a, Hadha, Ppara, and Slc25a20) in rats, whereas PRR significantly enhanced the expression of these four genes. Among these four genes, Cpt1a and Slc25a20 were reported to encode carnitine palmitoyltransferases 1 and carnitine acylcarnitine translocase, respectively, which can catalyze the transfer of long-chain fatty acids to the mitochondrial matrix in the form of acylcarnitines for fatty acid β-oxidation (Kerner and Hoppel, 2000). It is also known that inhibiting the expression of these two genes can induce the elevation of acylcarnitine (Longo et al., 2006). Interestingly, our study found that 19 acylcarnitines were significantly elevated by ANIT, whereas PRR could significantly reduce nine of these. Therefore, we concluded that PRR could enhance fatty acid β-oxidation. To our knowledge, this is the first report indicating that PRR attenuates cholestasis by alleviating the fatty acid β-oxidation dysfunction. It has been reported that the inhibition of fatty acid β-oxidation could increase the levels of serum fatty acids (Zhang et al., 2012). Thus, we inferred that the reduction of serum fatty acids by PRR may be through enhancing fatty acid β-oxidation in this study.

Moreover, according to the literature, the inhibition of fatty acid β-oxidation could result in lipid accumulation in hepatocytes and the subsequent production of reactive oxygen species, which contribute to hepatic inflammation and injury through the activation of Kupffer cells and hepatic stellate cells; the inhibition of fatty acid β-oxidation is considered a major mechanism of hepatotoxicity (Cusi et al., 2017). In this respect, several studies have reported that enhancing fatty acid β-oxidation could be considered as a therapeutic alternative for cholestasis (Zhao et al., 2017). Therefore, we believe that the enhancement of fatty acid β-oxidation can play an important role in the treatment of cholestasis by PRR.

### 4.3 The regulatory effects of PRR on gut microbiota may contribute to the reduction of secondary bile acids

To our knowledge, our study found for the first time that PRR could enrich the bacterial α diversity; and that the levels of five secondary bile acids downregulated by PRR were significantly negatively correlated with the relative abundance of four genera (*Coprococcus*, *Lactobacillus*, etc.) upregulated by PRR, and significantly positively correlated with the relative abundance of four genera (*Bacteroides*, *Escherichia*, etc.) downregulated by PRR, indicating that *Bacteroides*, *Escherichia*, *Turicibacter*, and *Allobaculum* might promote the production of secondary bile acids, but *Coprococcus*, *Lactobacillus*, *Ruminococcus*, and *Oscillospira* might inhibit the production of secondary bile acids.

It is well established that the gut microbiota participates in the production of secondary bile acids by controlling deconjugation, dehydrogenation, dehydroxylation and epimerization of primary bile acids in the distal small intestine and colon. It was reported that *Bacteroides* and *Escherichia* can express bile salt hydrolases (BSH) and 7α-hydroxysteroid dehydrogenases (7α-HSHD), respectively, which can catalyze the deconjugation and dehydrogenation of primary bile acids to promote the production of secondary bile acids (Gérard, 2014). And the elevation of the levels of *Bacteroides* and *Escherichia* will increase the expression and activity of BSH and 7α-HSHD in the intestine (Ovadia et al., 2020). Meanwhile, the increase in the levels of these two genera and the activity of BSH and 7α-HSHD causes the accumulation of secondary bile acids, which is one of the mechanisms of cholestatic liver diseases (Jiao et al., 2018). In our study, the relative abundance of *Bacteroides* and *Escherichia* was downregulated by PRR treatment and was positively correlated with the levels of five secondary bile acids downregulated by PRR, indicating that PRR may reduce the five secondary bile acids through the downregulation of *Bacteroides* and *Escherichia*. Whether the other six genera regulated by PRR observed in this study can express enzymes related to secondary bile acid metabolism has not been reported, but the relative abundance of these genera significantly correlated with the levels of secondary bile acids in the serum in our study. Thus, we concluded that the regulation of PRR on secondary bile acid metabolic disorder caused by ANIT was inseparable from its improvement of gut microbiota.

Moreover, it was reported that gut microbiota plays an important role in the progression of cholestasis by regulating metabolism and immune responses, so targeting the gut microbiota offers exciting new perspectives for the treatment of cholestatic liver diseases (Li et al., 2017). Based on this, we believe that the gut microbiota may be potential pharmacological target of the anti-cholestatic activity of PRR.

## Data Availability

The datasets presented in this study can be found in online repositories. The names of the repository/repositories and accession number(s) can be found below: NCBI BioProject, PRJNA866320.

## Abbreviations

PRR, Paeoniae Radix Rubra; ANIT, α-naphthyl isothiocyanate; UDCA, ursodeoxycholic acid; TCD, traditional Chinese drug; qRT-PCR, quantitative Real-Time PCR; ALP, alkaline phosphatase; AST, aspartate aminotransferase; ALT, alanine aminotransferase; TBA, total bile acid; TBIL, total bilirubin; DBIL, direct bilirubin; GGT, γ-glutamyl transpeptidase; H-E, hematoxylin and eosin; OTUs, operational taxonomic units; LDA, linear discriminant analysis; ANOVA, analysis of variance; PCA, principal component analysis; PLS-DA, partial least squares-discriminate analysis; MetPA, metabonomics pathway analysis; TGR5, G-protein-coupled receptor 5; FXR, farnesoid X receptor.

## References

[B1] Cai S. Y. Boyer J. L. (2021). The role of bile acids in cholestatic liver injury. Ann. Transl. Med. 9, 737–747. 10.21037/atm-20-5110 33987435 PMC8106037

[B2] Carbone M. Mells G. F. Pells G. Dawwas M. F. Newton J. L. Heneghan M. A. (2013). Sex and age are determinants of the clinical phenotype of primary biliary cirrhosis and response to ursodeoxycholic acid. Gastroenterology 144, 560–569. 10.1053/j.gastro.2012.12.005 23246637

[B3] Chascsa D. Carey E. J. Lindor K. D. (2017). Old and new treatments for primary biliary cholangitis. Liver Int. 37, 490–499. 10.1111/liv.13294 28371104

[B4] Cusi K. Bril F. Sunny N. E. (2017). Mitochondrial adaptation in nonalcoholic fatty liver disease: novel mechanisms and treatment strategies. Trends Endocrinol. Metab. 28, 250–260. 10.1016/j.tem.2016.11.006 27986466

[B5] Edgar R. C. (2013). UPARSE: highly accurate OTU sequences from microbial amplicon reads. Nat. Methods 10, 996–998. 10.1038/nmeth.2604 23955772

[B6] Fadrosh D. W. Ma B. Gajer P. Sengamalay N. Ott S. Brotman R. M. (2014). An improved dual-indexing approach for multiplexed 16S rRNA gene sequencing on the Illumina MiSeq platform. Microbiome 2, 6–12. 10.1186/2049-2618-2-6 24558975 PMC3940169

[B7] Gérard P. (2014). Metabolism of cholesterol and bile acids by the gut microbiota. Pathogens 3, 14–24. 10.3390/pathogens3010014 PMC4235735 25437605

[B8] He J. P. (2003). Research thoughts and methods of Chidan Tuihuang granule. Chin. J. Integr. Tradit. West. Med. Liver. 1, 52–53.

[B9] Holecek M. (2014). Evidence of a vicious cycle in glutamine synthesis and breakdown in pathogenesis of hepatic encephalopathy-therapeutic perspectives. Metab. Brain Dis. 29, 9–17. 10.1007/s11011-013-9428-9 23996300 PMC3930847

[B10] Houten S. M. Violante S. Ventura F. V. Wanders R. J. A. (2016). The biochemistry and physiology of mitochondrial fatty acid β-oxidation and its genetic disorders. Annu. Rev. Physiol. 78, 23–44. 10.1146/annurev-physiol-021115-105045 26474213

[B11] Hylemon P. B. Zhou H. Pandak W. M. Ren S. Gil G. Dent P. (2009). Bile acids as regulatory molecules. J. Lipid Res. 50, 1509–1520. 10.1194/jlr.R900007-JLR200 19346331 PMC2724047

[B13] Jiang Y. X. Li H. T. Song D. Ye P. H. Xu N. Chen Y. (2021). Comparative evidence for intrahepatic cholestasis of pregnancy treatment with traditional Chinese medicine therapy: a network meta-analysis. Front. Pharmacol. 12, 774884. 10.3389/fphar.2021.774884 34916949 PMC8670235

[B14] Jiao N. Baker S. S. Chapa-Rodriguez A. Liu W. S. Nugent C. A. Tsompana M. (2018). Suppressed hepatic bile acid signalling despite elevated production of primary and secondary bile acids in NAFLD. Gut 67, 1881–1891. 10.1136/gutjnl-2017-314307 28774887

[B15] Kerner J. Hoppel C. (2000). Fatty acid import into mitochondria. Biochim. Biophys. Acta 1486, 1–17. 10.1016/s1388-1981(00)00044-5 10856709

[B16] Kidd M. T. Kerr B. J. (1996). L-threonine for poultry: A review. J. Appl. Poult. Res. 5, 358–367. 10.1093/japr/5.4.358

[B17] Li Y. Tang R. Q. Leung P. S. C. Gershwin M. E. Ma X. (2017). Bile acids and intestinal microbiota in autoimmune cholestatic liver diseases. Autoimmun. Rev. 16, 885–896. 10.1016/j.autrev.2017.07.002 28698093

[B18] Liang J. Xu F. Zhang Y. Z. Huang S. Zang X. Y. Zhao X. (2013). The profiling and identification of the absorbed constituents and metabolites of Paeoniae Radix Rubra decoction in rat plasma and urine by the HPLC-DAD-ESI-IT-TOF-MS(n) technique: a novel strategy for the systematic screening and identification of absorbed constituents and metabolites from traditional Chinese medicines. J. Pharm. Biomed. Anal. 83, 108–121. 10.1016/j.jpba.2013.04.029 23727363

[B19] Lin S. Wang T. Y. Xu H. R. Xu X. R. Zhang Q. R. Liu R. (2019). A systemic combined nontargeted and targeted LC-MS based metabolomic strategy of plasma and liver on pathology exploration of alpha-naphthylisothiocyanate induced cholestatic liver injury in mice. J. Pharm. Biomed. Anal. 171, 180–192. 10.1016/j.jpba.2019.04.009 31009873

[B20] Liu X. J. Li H. W. Hu C. Wu L. J. Xiong Y. H. (2021). Evaluation of chemical liver injury based on metabolic profiling of serum fatty acids. J. Pharm. Sci. 56, 647–653.

[B21] Longo N. Amat di San Filippo C. Pasquali M. (2006). Disorders of carnitine transport and the carnitine cycle. Am. J. Med. Genet. C Semin. Med. Genet. 142, 77–85. 10.1002/ajmg.c.30087 PMC2557099 16602102

[B22] Luo Y. S. Gao F. Y. Chang R. R. Zhang X. J. Zhong J. Wen J. (2021). Metabolomics based comprehensive investigation of Gardeniae Fructus induced hepatotoxicity. Food Chem. Toxicol. 153, 112250–112264. 10.1016/j.fct.2021.112250 33964367

[B23] Ma X. Zhao Y. L. Zhu Y. Chen Z. Wang J. B. Li R. Y. (2015). *Paeonia lactiflora* Pall. protects against ANIT-induced cholestasis by activating Nrf2 via PI3K/Akt signaling pathway. Drug Des. Devel. Ther. 9, 5061–5074. 10.2147/DDDT.S90030 PMC4562737 26366057

[B24] Ma X. Chi Y. H. Niu M. Zhu Y. Zhao Y. L. Chen Z. (2016). Metabolomics coupled with multivariate data and pathway analysis on potential biomarkers in cholestasis and intervention effect of *Paeonia lactiflora* Pall. Front. Pharmacol. 7, 14–12. 10.3389/fphar.2016.00014 26869930 PMC4740759

[B25] Ma X. Wen J. X. Gao S. J. He X. Li P. Y. Yang Y. X. (2018). Paeonia lactiflora Pall. regulates the NF-κB-NLRP3 inflammasome pathway to alleviate cholestasis in rats. J. Pharm. Pharmacol. 70, 1675–1687. 10.1111/jphp.13008 30277564

[B26] Magoc T. Salzberg S. L. (2011). FLASH: fast length adjustment of short reads to improve genome assemblies. Bioinformatics 27, 2957–2963. 10.1093/bioinformatics/btr507 21903629 PMC3198573

[B27] Miyazaki T. Matsuzaki Y. (2014). Taurine and liver diseases: a focus on the heterogeneous protective properties of taurine. Amino Acids 46, 101–110. 10.1007/s00726-012-1381-0 22918604

[B28] Osena G. Nyaboga E. Amugune N. (2017). Rapid and efficient isolation of high quality DNA from cassava (Manihot esculenta Crantz) suitable for PCR based downstream applications. Annu. Res. Rev. Biol. 12, 1–10. 10.9734/arrb/2017/32195

[B29] Ovadia C. Perdones-Montero A. Fan H. M. Mullish B. H. Mcdonald J. A. K. Papacleovoulou G. (2020). Ursodeoxycholic acid enriches intestinal bile salt hydrolase-​expressing Bacteroidetes in cholestatic pregnancy. Sci. Rep. 10, 3895–3904. 10.1038/s41598-020-60821-w 32127609 PMC7054423

[B30] Pascale R. M. Simile M. M. Feo F. (2019). Alterations of methionine metabolism as potential targets for the prevention and therapy of hepatocellular carcinoma. Medicina 55, 296–319. 10.3390/medicina55060296 PMC6631235 31234428

[B31] Poland J. C. Flynn C. R. (2021). Bile acids, their receptors, and the gut microbiota. Physiology 36, 235–245. 10.1152/physiol.00028.2020 34159805 PMC8526331

[B32] State Pharmacopoeia Committee of People’s Republic of China (2020). Pharmacopoeia of people’s Republic of China. Beijing: China Medical Science and Technology Press.

[B33] Tanguay R. M. Angileri F. Vogel A. (2017). Molecular pathogenesis of liver injury in hereditary tyrosinemia 1. Adv. Exp. Med. Biol. 959, 49–64. 10.1007/978-3-319-55780-9_4 28755183

[B34] Tanner J. J. Fendt S. M. Becker D. F. (2018). The proline cycle as a potential cancer therapy target. Biochemistry 57, 3433–3444. 10.1021/acs.biochem.8b00215 29648801 PMC6026536

[B35] Wang L. L. Wu G. X. Wu F. H. Jiang N. Lin Y. N. (2017). Geniposide attenuates ANIT-induced cholestasis through regulation of transporters and enzymes involved in bile acids homeostasis in rats. J. Ethnopharmacol. 196, 178–185. 10.1016/j.jep.2016.12.022 27988401

[B36] Wang C. B. (2011). Integration of traditional Chinese and western medicine for diagnosing and treating sever Icteric liver disease. Beijing: People’s Military Medical Press.

[B37] Watanabe M. Houtenl S. M. Matakil C. Christoffolete M. A. Kim B. W. Satol H. (2006). Bile acids induce energy expenditure by promoting intracellular thyroid hormone activation. Nature 439, 484–489. 10.1038/nature04330 16400329

[B38] Waters N. J. Holmes E. Williams A. Waterfield C. J. Farrant D. Nicholson J. K. (2001). NMR and pattern recognition studies on the time-related metabolic effects of α-naphthylisothiocyanate on liver, urine, and plasma in the rat: an integrative metabonomic approach. Chem. Res. Toxicol. 14, 1401–1412. 10.1021/tx010067f 11599932

[B39] Wei S. S. Zhao Y. L. Jiang F. J. Jia L. Zhu Y. Wang J. B. (2011). Dose-effect-response relationships of Paeoniae Radix Rubra on α-naphthylisothiocyanate-induced acute cholestatic hepatitis in rats. Chin. Herb. Med. 3, 296–303.

[B40] Zhang Y. Y. Li F. Patterson A. D. Wang Y. Krausz K. W. Neale G. (2012). Abcb11 deficiency induces cholestasis coupled to impaired β-fatty acid oxidation in mice. J. Biol. Chem. 287, 24784–24794. 10.1074/jbc.M111.329318 22619174 PMC3397905

[B41] Zhao Q. Yang R. Wang J. Hu D. D. Li F. (2017). PPARα activation protects against cholestatic liver injury. Sci. Rep. 7, 9967–9979. 10.1038/s41598-017-10524-6 28855630 PMC5577315

