# Supplementary material for: Paeoniae Radix Rubra can enhance fatty acid β-oxidation and alleviate gut microbiota disorder in α-naphthyl isothiocyanate induced cholestatic model rats
Source: Front Pharmacol. 2022 Oct 21;13:1002922. doi: 10.3389/fphar.2022.1002922 (PMC9633937; doi:10.3389/fphar.2022.1002922)
Supplement: Supplementary file 1 [file DataSheet1.docx]

Supplementary Information 1

Chemical fingerprint of the PRR extract

To elucidate the chemical features of the PRR extract we used in this study, an UFLC method was established to determine its chemoprofile.

# Preparation of samples

32.8 mg PRR freeze-dried powder was dissolved in 10 mL water with ultrasound, then centrifuged at 8000 rpm for 10 min and the supernatant was filtered and injected for analysis.

# UFLC-based chemoprofile of the PRR extract

The separation was performed on a SHIMAZDU LC-30A System (Shimazdu Corp., Japan) using an Acquity UPLC HSS T3 column (100×2.1 mm, i.d., 1.8 μm, Waters, USA). 2 μL of sample solution was injected into system and the column temperature was maintained at 25℃. The wavelength was set at 275 nm. The mobile phase was composed of 0.1% formic acid aqueous solution (A) and acetonitrile (B) at a flow rate of 0.3 mL/min using a linear gradient elution program shown in Table S1.

Some main constituents of PRR extract were identified based on the retention time, and mass spectrometry fragmentations. Mass spectrographic analysis was performed using Q Exactive mass spectrometer (Thermo Fisher Scientific, Massachusetts, USA) in negative electrospray ionization mode. The scan range was m/z 70~1000, with a spray voltage of 3700V, a capillary temperature of 320°C, a flowrate of sheath gas at 28 L·h^-1^, and a flowrate of aux gas at 8 L·h^-1^. Capillary voltage and cone hole voltage were set at 2300 V and 40 V, respectively.

The UFLC-based chemoprofile of the PRR extract was shown in Figure S1.

**Table S1 UFLC gradient program of the PRR extract analysis**

| **Time (min)** | **Mobile phase B%** |
| --- | --- |
| 0–20 | 5–20 |
| 20–30 | 20–30 |
| 30–40 | 30–55 |
| 40–50 | 55–60 |
| 50–65 | 60–100 |


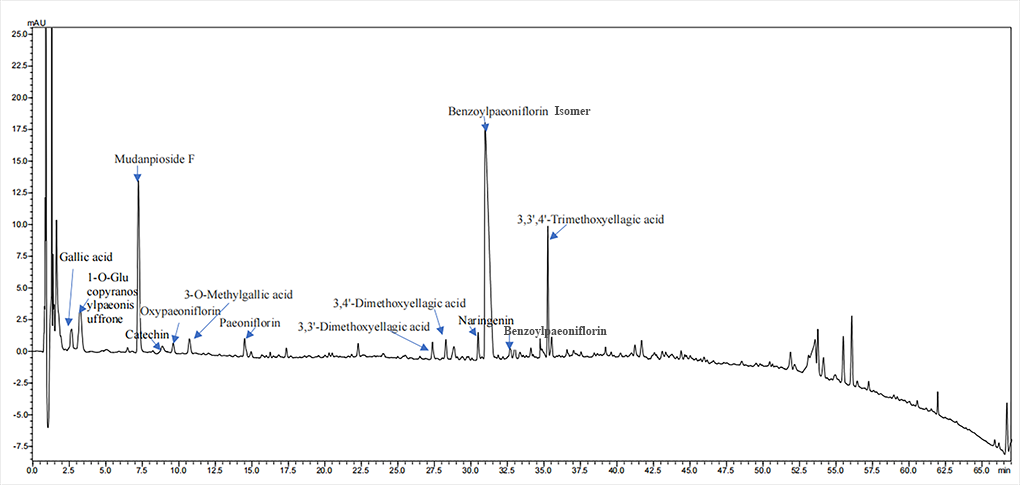


**Figure S1 Chemical fingerprint of the PRR extract using UFLC**

Supplementary Information 2

Determination of 5 major constituents in the PRR extract

To further ensure the reproducibility of the animal experiment results in this study, a UFLC-MS/MS method of simultaneous analysis of 5 major constituents (paeoniflorin, catechin, oxypaeoniflorin, benzoylpaeoniflorin, gallic acid) in the PRR was established and validated. Meanwhile, their contents in the PRR extract used in this study were measured.

# Materials and methods

# 1.1 Reagents and chemicals

Reference substances of paeoniflorin (PS000825), oxypaeoniflorin (PS010199), benzoylpaeoniflorin (PS000158) were purchased from Chengdu Push Bio-technology Co., Ltd (Chengdu, China). Catechin (A0158) and gallic acid (A0110) were purchased from Chengdu Must Biotechnology Co. Ltd (Chengdu, China).

LC-MS grade acetonitrile and HPLC grade methanol were purchased from Fisher Scientific (Fair Lawn, NJ, USA). Ultra-high purity water (18.2 MΩ, TOC< 5 ppb) was prepared by a Millipore Milli-Q Integral 3 Ultrapure Water System (Billerica, MA, USA). HPLC grade formic acid was purchased from Tedia Company Inc. (Fairfield, OH, USA). Other chemicals were all analytical reagents.

PRR was purchased from the Beijing Tianheng pharmacy (Beijing, China; Lot no. 14701), and it was authenticated as the dried roots of *Paeonia lactiflora* Pall. by Dr. Feng Xu (School of Pharmaceutical Sciences, Peking University). A voucher sample (No. 7838) was deposited in the Herbarium of Pharmacognosy, School of Pharmaceutical Sciences, Peking University.

# 1.2 Preparation of standard solutions

Stock standard solutions of the 5 constituents were prepared with methanol and then diluted to different concentrations. All the stock standard solutions were stored at 4 °C in the refrigerator.

**1.3 LC-MS instruments and operation conditions**

**1.3.1 Liquid chromatography**

The separation was performed on an SHIMAZDU LC-30A System (Shimazdu Corp., Japan) using an Acquity UPLC HSS T3 column (100×2.1 mm, i.d., 1.8 μm, Waters, USA). A 2 μL sample was injected into system and the column temperature was maintained at 35°C. The mobile phase was composed of 0.1% formic acid aqueous solution (A) and acetonitrile (B) at a flow rate of 0.35 mL/min using a gradient elution of 15% B at 0–0.5 min, 15%–50% B at 0.5–3.5 min, 50%–90% B at 3.5–6.5 min, 90% B at 6.5–7 min, 90%–15% B at 7–7.5 min.

**1.3.2 Mass spectrometric conditions**

An 8050 triple quadrupole mass spectrometer (Shimadzu Corp., Japan) equipped with an electrospray ionization (ESI) interface was used for analytical detection. ESI/MS was operated in negative mode. The operating MS parameters were as follows: drying gas (N2) ﬂow rate, 10.0 L/min; nebulizing gas ﬂow rate, 3.0 l/min; heating gas ﬂow rate, 10.0 L/min; interface voltage, 3 kV; detector voltage, 1.8 kV; interface temperature, 300 °C; desolvation temperature, 250 °C; heat block temperature, 400 °C.

# Preparative method of PRR sample

32.8 mg PRR freeze-dried powder was dissolved in 10 mL water with ultrasound, then centrifuged at 8000 rpm for 10 min and the supernatant was filtered and injected for analysis.

# Method validation

**3.1 Linearity**

The linearity was assessed by assaying calibration curves with standard work solutions. Peak areas of analytes were recorded and calibration curves were drawn, then regression equations of the curves and correlation coefficients (r^2^) were calculated.

**3.2 Precision**

The precisions at three levels were evaluated by using the high-, medium- and low-level quality control samples prepared above.

**3.3 Stability**

Three concentrations of samples were assayed at 4, 12 and 24 h after the PRR extract was prepared. The peak areas of analytes were recorded. The averages and standard deviation (SD) values of concentrations for 5 constituents in 24 h were calculated and relative standard deviation (RSD%) was also calculated as an estimate of stability.

# Results

**4.1 Method validation**

The calibration curves, correlation coefficients, linear ranges, high-, medium-, low-concentration precision and stability within 24 h of the 5 analytes are showed in Tables S2 and S3. The calibration curves of 5 analytes exhibited good linearity within the selected ranges with the correlation coefficients (r2) between 0.997 and 0.999. All the results indicated that the established method had a satisfactory precision and stability.

**4.2 Contents of 5 constituents in the PRR extract**

The prepared sample of the PRR extract was injected and analyzed for 3 times. The areas of 5 analytes were recorded and contents were calculated with regression equations.

The contents of 5 analytes in the PRR extract were as follows: paeoniflorin 5.63 mg/g, catechin 0.13 mg/g, oxypaeoniflorin 0.41 mg/g, benzoylpaeoniflorin 0.13 mg/g, gallic acid 0.15 mg/g.

**Table S2 Molecular formula, quantification transition, retention time, calibration curve and correlation coefficient of 5 constituents in the PRR extract**

| **Constituent** | **Molecular formula** | **Quantification transition (m/z)** | **Retention time (t_R_, min)** | **Calibration curve** | **Correlation coefficient (r^2^)** |
| --- | --- | --- | --- | --- | --- |
| **Paeoniflorin** | C_23_H_27_O_11_ | 525.30>449.05 | 2.50 | y = 3.51×10^6^x + 1.57×10^6^ | 0.999 |
| **[Catechin](http://www.baidu.com/link?url=XBgxXrWgdD3ul4mSwFRIApbHITHtHsqO-J70JFcsAWgD__pLWnx35TFQ869zvt7yw9_ePcI4_JsXOg8W4uDytxUH5ps_qlkHIocCBSBhcC3)** | C_15_H_13_O_6_ | 289.20>245.10 | 1.85 | y = 1.91×10^6^x – 639.08 | 0.997 |
| **Oxypaeoniflorin** | C_23_H_27_O_12_ | 495.20>137.10 | 1.50 | y = 3.95×10^6^x + 189.21 | 0.999 |
| \|  \| [**Benzoylpaeoniflorin**](http://www.baidu.com/link?url=-Hg5ffzHsQF9FEMcivdufNNK-qIuaAnb9jWBPeJlDC_Sx2iV4jUlh2TS9ccZ5VCpEarUs0BVh-5zcCSVBqwkNcG1SJejpCaeYjNQJRxeHRhfhOE3Am5uCm6Zcq7nRvt5) \| \| --- \| --- \| | C_30_H_31_O_12_ | 629.35>121.05 | 3.98 | y = 3.76×10^6^x + 38.75 | 0.999 |
| **[Gallic acid](http://www.baidu.com/link?url=eISCTQ2lfSLeFgAzdQFVy1X_qpFmlD9Nl5DRokvcQGHDoNZ2ClSWNSoGw78Vb28pGHd0aGBEYZxBO97ICn9bqvGWFcQJLlKk_JupL4CGSC_)** | C_7_H_5_O_5_ | 169.20>125.05 | 1.02 | y = 635.32x + 55.48 | 0.999 |

**Table S3 Linear ranges, high-, medium- and low-concentration precisions and stability in 24 h of 5 constituents in the PRR extract**

| **Constituent** | **Linear ranges**  **(μg/ml)** | **Precisions** | | | | | | | **Stability in 24 h**  **(RSD)** |
| --- | --- | --- | --- | --- | --- | --- | --- | --- | --- |
|  |  | **High (ng/ml, %)** | | | **Medium (ng/ml, %)** | | **Low (ng/ml, %)** | |  |
| **Paeoniflorin** | 0.7812–25.00 | 12.50 | 99.78 | 3.125 | | 102.87 | 0.7812 | 99.20 | 1.14 |
| [**Catechin**](http://www.baidu.com/link?url=XBgxXrWgdD3ul4mSwFRIApbHITHtHsqO-J70JFcsAWgD__pLWnx35TFQ869zvt7yw9_ePcI4_JsXOg8W4uDytxUH5ps_qlkHIocCBSBhcC3) | 0.01560–0.5000 | 0.2500 | 104.40 | 0.06250 | | 100.27 | 0.01560 | 91.74 | 3.79 |
| **Oxypaeoniflorin** | 0.08825–2.825 | 1.413 | 103.50 | 0.3531 | | 103.55 | 0.8825 | 103.08 | 0.16 |
| **Benzoylpaeoniflorin** | 0.03280–1.050 | 0.5250 | 100.43 | 0.1312 | | 100.83 | 0.03280 | 94.51 | 0.74 |
| [**Gallic acid**](http://www.baidu.com/link?url=eISCTQ2lfSLeFgAzdQFVy1X_qpFmlD9Nl5DRokvcQGHDoNZ2ClSWNSoGw78Vb28pGHd0aGBEYZxBO97ICn9bqvGWFcQJLlKk_JupL4CGSC_) | 0.1625–5.200 | 2.600 | 100.81 | 0.6500 | | 100.02 | 0.1625 | 99.49 | 4.17 |

***Supplementary Information 3***

**Table S4 Effects of PRR on serum ALT, AST, ALP, GGT, TBIL, DBIL, and TBA in ANIT induced cholestatic model rats (n=3)**

| **Group** | **ALT(U/L)** | **AST(U/L)** | **ALP(U/L)** | **GGT(U/L)** | **TBIL(μmol/L)** | **DBIL(μmol/L)** | **TBA(μmol/L)** |
| --- | --- | --- | --- | --- | --- | --- | --- |
| **Control** | 71.00±5.29 | 116.67±15.04 | 399.67±74.22 | 1.00±0.00 | 1.37±0.06 | 0.17±0.29 | 27.73±6.35 |
| **Model** | 2499.33±337.51^##^ | 3054.00±543.04^##^ | 993.00±124.05^##^ | 8.33±2.31^##^ | 146.63±7.92^##^ | 122.33±6.11^##^ | 365.83±38.84^##^ |
| **PRR** | 646.33±66.94^**^ | 1293.67±270.99^**^ | 843.67±150.53^**^ | 7.33±1.15^*^ | 117.70±39.09^**^ | 94.43±34.30^**^ | 307.90±34.92^**^ |
| **UDCA** | 463.67±132.73^**^ | 735.33±183.22^**^ | 732.00±140.99^**^ | 6.67±2.08^**^ | 91.13±1.59^**^ | 72.17±0.91^**^ | 281.77±41.30^**^ |

^##^p<0.01 versus control group. *p<0.05, **p<0.01 versus model group.

**Table S5 The primers’ sequences used in RT-qPCR**

| **Gene** | **Sequence** | **Annealing temperature (℃)** | **Product length (bp)** |
| --- | --- | --- | --- |
| GAPDH(RAT) | F:5’ GCTCTCTGCTCCTCCCTGTTCTA3’  R:5’ TGGTAACCAGGCGTCCGATA3’ | 60 | 124 |
| Cpt1a | F:5’ AGAGACAGACACCATCCAACAT3’  R:5’ TAGAGCCAGACCTTGAAGTACC3’ | 60 | 84 |
| Ehhadh | F:5’ ATTGTGATCTGTGGCGCAAAC 3’  R:5’ ACACCGGCTTCTGGTATCTC 3’ | 60 | 127 |
| Hadha | F:5’ AAAGCCTGGCTGCTTTGTTG 3’  R :5’ CCGGCTCCTGGTAAGATTCC 3’ | 60 | 270 |
| Acat2 | F:5’ CTCGGAGCTACAGGATGAATGC 3’  R:5’ TAACAGTTGTCCCCAGGTT 3’ | 60 | 126 |
| Ppara | F:5’ GCCGTTTCCACAAGTGCC 3’  R :5’ CTTTCCTGCGAGTATGACCC 3’ | 60 | 230 |
| Slc25a20 | F:5’ GCGGAGCAAGATCAAGACTG 3’  R :5’ CAGGCAAACTTGGTGGTTGT 3’ | 60 | 194 |

**Table S6** Significantly changed fatty acids in the liver tissue between the model group and control group or between PRR group and model group

| **No.** | **Metabolites** | **Model group/Control group** | **PRR group/Model group** |
| --- | --- | --- | --- |
| 1 | FFA 20:3 ^a^ | ↑** | ↓** |
| 2 | FFA 22:4 ^a^ | ↑** | ↓** |
| 3 | FFA 22:5 ^a^ | ↑** | ↓** |
| 4 | FFA 18:0 ^a^ | ↑** | ↓** |
| 5 | FFA 20:4 ^a^ | ↑** | ↓** |
| 6 | FFA 16:0 ^a^ | ↑** | ↓** |
| 7 | FFA 18:1 ^a^ | ↑** | ↓** |
| 8 | FFA 18:2 ^a^ | ↑** | ↓** |
| 9 | FFA 16:0 ^a^ | ↑** | ↓** |

**p<0.05*; ***p<0.01*; ^a^ for the name of fatty acids, the number before the colon represents the number of carbon atoms; the number behind the colon represents the number of double bonds.

***Supplementary Information 4***

**Targeted metabonomics analysis methods**

# Analysis of bile acids

UPLC separation was performed with an ACQUITY UPLC I-Class System (fixed loop), equipped with a CORTECS T3 2.7 μm (2.1 × 30 mm) analytical column. A sample of 1 μL was injected at a flow rate of 1.3 mL/min. Mobile phase A was 0.01% formic acid containing 0.2 mmol/L ammonium formate and mobile phase B was 50% isopropanol in acetonitrile containing 0.01% formic acid and 0.2 mmol/L ammonium formate. The gradient elution was performed as follows: 0–0.7 min, 20–55% B; 0.7–0.9 min, 55%–98% B. The column was then re-equilibrated to initial conditions. The analytical column temperature was maintained at 60 ℃.

Multiple Reaction Monitoring analyses were performed using a Xevo TQ-S micro mass Spectrometer. All experiments were performed in negative electrospray ionization (ESI-) mode. The ion source temperature and capillary voltage were kept constant and set to 150℃ and 2.0 kV respectively. The cone gas flow rate was 50 L/hr and desolvation temperature was 650 ℃.

# Analysis of fatty acids

UPLC separation was performed with an ACQUITY UPLC I-Class System (fixed loop), equipped with a CORTECS T3 2.7 μm (2.1 × 30 mm) analytical column. A sample of 2 μL was injected at a flow rate of 1.3 mL/min. Mobile phase A was 0.01% formic acid containing 0.2 mmol/L ammonium formate and mobile phase B was 50% isopropanol in acetonitrile containing 0.01% formic acid and 0.2 mmol/L ammonium formate. The free fatty acids were eluted from the column and separated with a gradient of 50-98% mobile phase B over 1.2 minutes followed by a 0.5 minutes column wash at 98% mobile phase B. The column was then re-equilibrated to initial conditions. The analytical column temperature was maintained at 60 ℃.

Multiple Reaction Monitoring analyses were performed using a Xevo TQ-S micro mass Spectrometer. All experiments were performed in positive electrospray ionization (ESI+) mode. The ion source temperature and capillary voltage were kept constant and set to 150℃ and 2.0 kV respectively. The cone gas flow rate was 50 L/hr and desolvation temperature was 650 ℃.

# Analysis of amino acids

The analysis was performed using a Shimadzu LC 20 AD system (Shimadzu, Kyoto, Japan) connected to a 5500 Q-trap mass spectrometer (AB Sciex, Redwood, USA). Separation was performed on an Acquity BEH amide column (1.7 mm × 100 mm, 1.8 μm) kept at 50◦C and at a flow rate of 0.3 mL/min. The gradient mobile phase was a mixture of 0.1% formic acid and 2.5 mmol/L ammonium formate in water (A) and acetonitrile (B). The gradient elution was performed as follows: 0–6.0 min, 75% B; 6.0–15.0 min, 75%–100% B. 1 μL of sample solution was injected for each run. During the whole analysis, all the samples were maintained at 15◦C. The curtain gas was 40 kPa, collision gas was medium, ionspray voltage was 1500 V, nebulizer gas was 50 kPa, heater gas was 60 kPa, declustering potential was 10 V and ionization temperature was 600◦C. The scan was in negative mode and the scan range was m/z 100–1500.

# Analysis of organic acids

UPLC separation was performed with an ACQUITY UPLC I-Class System (fixed loop), equipped with a ACQUITY UPLC BEH C8 1.7 μm (2.1 × 100 mm) analytical column. A sample of 1 μL was injected at a flow rate of 0.3 mL/min. Mobile phase A was 0.01% formic acid and mobile phase B was 20% isopropanol in methanol. The gradient elution was performed as follows: 0–2.0 min, 5%–15% B; 2.0–9.0 min, 15%–55% B; 9.0–10.0 min, 55%–100% B. The analytical column temperature was maintained at 45 ℃.

Multiple Reaction Monitoring analyses were performed using a Xevo TQ-S micro mass Spectrometer. All experiments were performed in negative electrospray ionization (ESI-) mode. The ion source temperature and capillary voltage were kept constant and set to 150℃ and 3.0 kV respectively. The cone gas flow rate was 100 L/hr and desolvation temperature was 500 ℃.

# Analysis of acylcarnitines

UPLC separation was performed with an ACQUITY UPLC I-Class System (fixed loop), equipped with a CORTECS T3 2.7 μm (2.1 × 30 mm) analytical column. A sample of 0.5 μL was injected at a flow rate of 1.3 mL/min. Mobile phase A was 0.01% formic acid containing 0.2 mmol/L ammonium formate and mobile phase B was 50% isopropanol in acetonitrile containing 0.01% formic acid and 0.2 mmole ammonium formate. After an initial 0.1 minutes hold at 2% mobile phase B, the acylcarnitines were eluted from the column and separated with a gradient of 2-98% mobile phase B over 0.7 minutes, followed by a 0.9 minutes column wash at 98% mobile phase B. The column was then re-equilibrated to initial conditions. The analytical column temperature was maintained at 60 ℃.

Multiple Reaction Monitoring analyses were performed using a Xevo TQ-S micro mass Spectrometer. All experiments were performed in positive electrospray ionization (ESI+) mode. The ion source temperature and capillary voltage were kept constant and set to 150℃ and 2.0 kV respectively. The cone gas flow rate was 50 L/hr and desolvation temperature was 650 ℃.
